# Interactive Knowledge-Based Kernel PCA for Solvent Selection

**DOI:** 10.1021/acssuschemeng.4c07974

**Published:** 2025-03-14

**Authors:** Samuel Boobier, Joseph Heeley, Thomas Gärtner, Jonathan D. Hirst

**Affiliations:** † School of Chemistry, University of Nottingham, University Park, Nottingham, NG7 2RD, U.K.; ‡ Machine Learning Research Unit, TU Wien Informatics, Vienna 1040, Austria

**Keywords:** solvent selection, machine learning, interactive visualization, green chemistry, principal component analysis, open source, electronic laboratory notebook

## Abstract

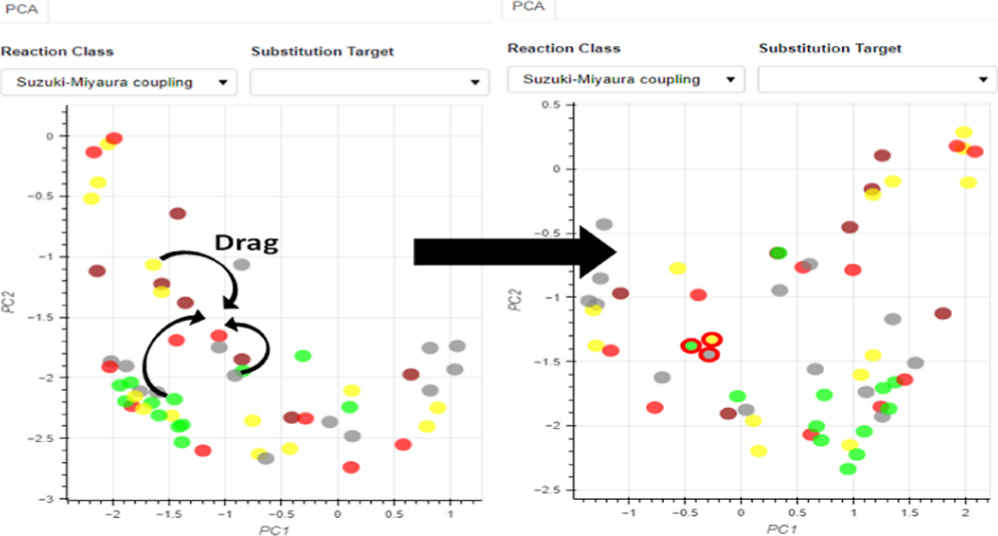

Selecting more sustainable solvents is a crucial component to mitigating the environmental impacts of chemical processes. Numerous tools have been developed to address this problem within the pharmaceutical industry, employing data-driven approaches such as multidimensional scaling or principal component analysis (PCA). Interactive knowledge-based kernel PCA is a variant of PCA that allows users to shape 2D solvent maps by defining the positions of data points, imparting expert knowledge that was not included in the original descriptor set. We have applied interactive PCA to the task of solvent selection and present an intuitive interface that is integrated into AI4Green, an electronic laboratory notebook that encourages sustainable chemistry. A set of evidence-based user guidelines were developed and used in combination with the interactive PCA to identify four potential solvent substitutions for an example thioesterification reaction.

## Abstract 2

The Solvent Surfer helps the user to identify solvent alternatives by allowing users to interact directly with PCA data visualization.

## Introduction

Sustainability is an important concept in the chemical sciences, where high energy demands and the generation of volumes of waste have dangerous implications for health, safety, and the environment.^1,2^ The advent of the 12 principles of green chemistry in 1998^3^ signified a paradigm shift toward environmental awareness and away from unsustainable practices, resulting in a range of industrial initiatives being implemented to address and mitigate the environmental impact of chemistry.^4−6^ These initiatives remain, for the most part, focused on the large-scale manufacture of chemicals.^7^ The reasons for this are seemingly obvious; the negative impacts of chemistry are more severe at scale and mitigating these leads to the highest pay-off. This is neither a sustainable nor preventative approach, as environmentally harmful decisions made at discovery level must be mitigated prior to scale-up. While this continues to be the case, the implementation of green initiatives at process will remain a challenge.^8^

Addressing this problem was the main drive behind the development of AI4Green,^9^ an electronic laboratory notebook (ELN) that places an emphasis on sustainable chemistry. Digital tools that increase awareness of unsustainable practices are integrated into the ELN, allowing users to assess the sustainability of their chemistry and easily make more sustainable choices. An example of such a digital tool is the AI4Green solvent guide, which provides an intuitive interface for the identification and comparison of solvent “greenness” data. As solvents account for more than half of the waste produced during the development of active pharmaceutical ingredients,^10^ selecting more sustainable solvents can have a big impact upon the environmental implications of a given process. For this reason, a range of paper-based selection guides have been developed for use in the pharmaceutical industry,^11−13^ though surveys of these guides have found that conflicting information is provided in some cases.^14,15^ In 2016, the Pharmaceutical Roundtable Innovative Medicines Initiative (IMI-CHEM21) published a heuristic selection guide that aimed to evaluate solvents with reference to the Globally Harmonised System (GHS) for labeling chemical hazards,^16^ ranking each solvent as either “Recommended”, “Problematic”, “Hazardous” or “Highly Hazardous”. The AI4Green solvent guide provides an interface for the visualization of these results and has been recently released as a standalone package.^17^

While these solvent selection guides provide an easy method for identifying greener solvents, other methods are data-driven. For example, the solubility parameter (δ), introduced by Hansen in 1967,^18,19^ has been used to identify potential solvents for polymers by considering a combination of dispersion (δ*D*), polarity (δ*P*) and hydrogen-bonding (δH) interactions. Solvents with similar parameters are more likely to exhibit similar behaviors and comparing these can allow the identification of alternatives. Similarly, the Kamlet–Abboud–Taft parameters describe solvents in terms of their polarizability (π*), hydrogen-bond acidity (α) and hydrogen-bond basicity (β),^20,21^ and can be used to predict the linear free-energy of solvation for various solvent–solute pairings.

More recently, solvent selection tools have exploited techniques such as multidimensional scaling (MDS) or principal component analysis (PCA).^22,23^ These techniques are used to reduce complex multidimensional data sets into two-dimensional representations that are simpler and easier to visualize. These two-dimensional representations or “embeddings” are obtained using statistical transformations that retain important information from the initial data set.

The Sustainable Solvents Selection and Substitution Software (SUSSOL)^24^ applies MDS to a data set of 500 solvents described by 22 physical properties to map solvent space. The PCA example, initially developed by AstraZeneca^25^ and currently hosted on the ACS Web site,^26^ provides a dimensionality reduction of 272 solvents described by 70 experimental and calculated physical properties. Both examples show that the generated solvent maps accurately reflect external experimental data and demonstrate the next generation of intelligent solvent selection tools, though many other studies have been undertaken.^27−30^

However, the lower dimensional embeddings returned from MDS or PCA are inherently static and cannot be easily tailored for a specific use-case. Interactive knowledge-based kernel PCA^31−33^ instead allows users to explore different embeddings by clicking and dragging data points to new positions within the visualization based on real-world experimental data, triggering an automatic recalculation that reflects these changes. Any data used to group these “control points” are thus reflected in the updated embeddings, providing more specialized insights into domain-specific applications than the previous static embeddings.

The application of interactive knowledge-based kernel PCA to the task of solvent selection as a sustainability plug-in for AI4Green provides users with a data-driven and customizable approach for identifying solvent alternatives. It is envisaged that solvents could be grouped according to some response from real experimental data, the nature of which would be dependent on the task at hand; percentage yields, conversions, reaction rates, or selectivities would be beneficial for solvent screening while compound solubilities would be preferred for identifying cosolvents for crystallization. These data could be used to identify various “activity domains” by which to group the solvents, providing information about a solvent’s propensity toward a specific task and subsequently reflecting this in the updated embedding. These newly defined areas could then be interrogated to identify solvents close by that could act as alternatives. This user-guided customization offers a significant advantage over the static one-time PCA maps, which cannot be altered to reflect specific use-cases.

## Methods

### Incorporating Knowledge-Based Constraints

This section introduces the optimization problem that must be solved to account for user inputs in kernel PCA.^31^ This optimization seeks to identify orthogonal principal components that maximize the variance of the data while including constraints provided by the user. For the task of solvent selection, these constraints are provided by directly interacting with the PCA visualization to define the position of control points that are used to update the embedding.

For a given sample Χ = {*x*_1_, ..., *x*_*n*_} and a reproducing kernel Hilbert space, , with kernel *k*(·, ·) and  the directions of the -norm maximum variance (or the principal components) can be expressed as
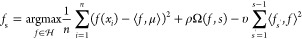
1subject to
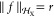
where Ω is the term for the inclusion of control points and . The control point term is included as
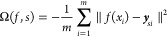
2where *y*_*si*_ is the new coordinate of the control point along the projection axis *f*_*s*_ for a given example *x*_*i*_. The weak representer theorem^34,35^ holds for both eq 1 and 2, such that *f*_*s*_ = ∑_*i*_α_*si*_*k*(*x*_*i*_, ·) where , so eq 1 can be reformulated as
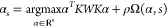
3subject to
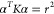
where , *H*_*n*_ = *I*_*n*_-*ee*^*T*^, *I*_*n*_ is the identity matrix, e is a vector of ones, *K* is the kernel matrix and eq 2 becomes
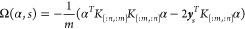
4

Rewriting the optimization problem in terms of the kernel matrix significantly reduces the computational complexity of this approach. The Electronic Supporting Information details how further improvements can be made to reduce the computational complexity.

### Data Set

The data set generated for the PCA contains 57 solvents, each described by a set of 16 descriptors. The list of solvents includes those that are commonly used during pharmaceutical research such as dichloromethane and 1,4-dioxane. Additional alternative solvents, including γ-valerolactone (GVL) and propylene carbonate, are also included. Each solvent was assigned a CHEM21 score where possible^16^ and the associated cost is included in GBP/Liter as determined by Sigma-Aldrich. These values are intended as an approximate guide and do not update to reflect the current state of the market. For accurate costs, users should visit appropriate vendor Web sites.

The descriptors were chosen to convey the overall properties of the solvents and are summarized in Table 1. The physical attributes of a solvent are important to consider when evaluating potential substitutions as they can have significant practical implications. Descriptors that capture these properties, such as boiling point, molecular weight, density, viscosity, molar volume, vapor pressure and refractive index were included. The dielectric constant and dipole moment were also added to provide a description of polarity, a vital consideration for solvent selection and substitution. The octanol: water partition coefficient (Log *P*), which describes the hydrophobicity of a solvent, is often used as an orthogonal measure for polarity and was included on this basis. As both Hansen and Kamlet–Abboud–Taft parameters have been previously shown to be useful in predicting solubilities, they were included in the data set as a description of the chemical attributes of the solvents. Full details on the consolidation of these data and the full solvent list are available in the Supporting Information.

**Table 1:** Summary of the Full Descriptor Set Used for PCA

descriptor	units	mean	St Dev	range
molecular weight	g mol^–1^	91.36	29.68	18.02–179.20
boiling point	°C	119.66	59.52	34.60–248.00
density	g mL^–1^	0.99	0.22	0.63–1.59
viscosity	cP	1.71	2.62	0.22–1 6.10
molar volume	mL mol^–1^	93.88	26.83	18.12–173.98
vapor pressure	mmHg	75.24	117.20	0.00–538.00
refractive index		1.41	0.06	1.28–1.56
dielectric constant		18.35	19.16	1.80–89.78
dipole moment	Debye	2.07	1.39	0.00–4.77
Log P		0.80	1.45	–1.38–4.66
Hansen δ*D*	MPa1/2	16.78	1.50	14.50–20.00
Hansen δP	MPa1/2	7.26	5.20	0.00–21.70
Hansen δH	MPa1/2	8.57	7.34	0.00–42.30
Kamlet–Abboud–Taft α		0.26	0.44	0.00–1.96
Kamlet–Abboud–Taft β		0.41	0.27	0.00–1.05
Kamlet–Abboud–Taft π*		0.62	0.29	–0.08–1.09

### Implementation

The backend of the AI4Green web application is written in Python and leverages the Flask blueprint framework. Flask blueprints encourage modular application design and allow for easy extraction/addition of key application features. The code for the interactive PCA is also written in Python and was adapted into a Flask blueprint from the InVis software,^32,33^ allowing for easy integration into AI4Green. A Gaussian kernel was used for all PCAs. The interface developed for AI4Green is written in Javascript, HTML and CSS. The data set and source code are publicly available on the AI4Green GitHub: https://github.com/AI4Green/AI4Green/tree/main/Webapp/sources/services/solvent_surfer. The Solvent Surfer interface can be accessed as part of AI4Green at: https://ai4green.app/solvent_PCA. Login is required to access this feature.

## Results and Discussion

### Interactive PCA

**Figure 1:**
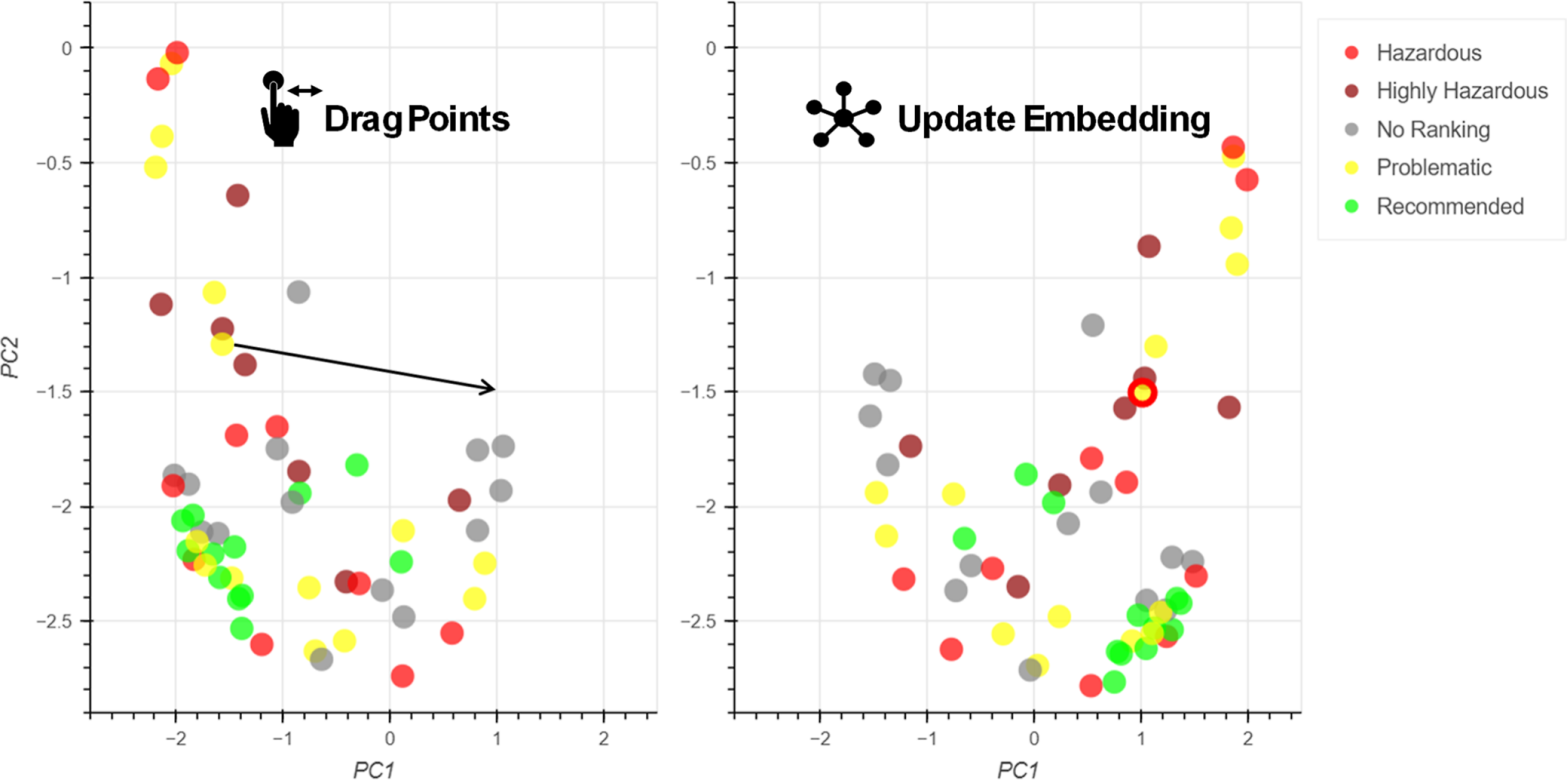
Interactive knowledge-based kernel PCA of the complete solvent data set. Points can be moved by clicking and dragging and the embedding is updated when they are dropped. Colors correspond to the CHEM21 rankings of the solvents.

The main functionality for the interactive knowledge-based kernel PCA is the same as the InVis software;^32,33^ users can define the position of control points by clicking and dragging them into place and the positions of the remaining points are automatically updated (Figure 1). This allows the embedding to evolve alongside chemical discovery, providing a more useful guide for the selection of solvent alternatives.

For this tool to be widely adopted, a set of clear and evidence-based instructions are needed to guide users toward the best practices. To develop such guidelines, it was crucial to understand how solvents should be selected as control points and how their movements affect the resultant embeddings. It was also important to identify the optimal number of control points and investigate whether the same embedding could be achieved if control points were grouped in different areas of the 2D space. Considering these three factors would provide an in-depth assessment of the interactive solvent PCA and forms the basis for the following investigation.

**Figure 2:**
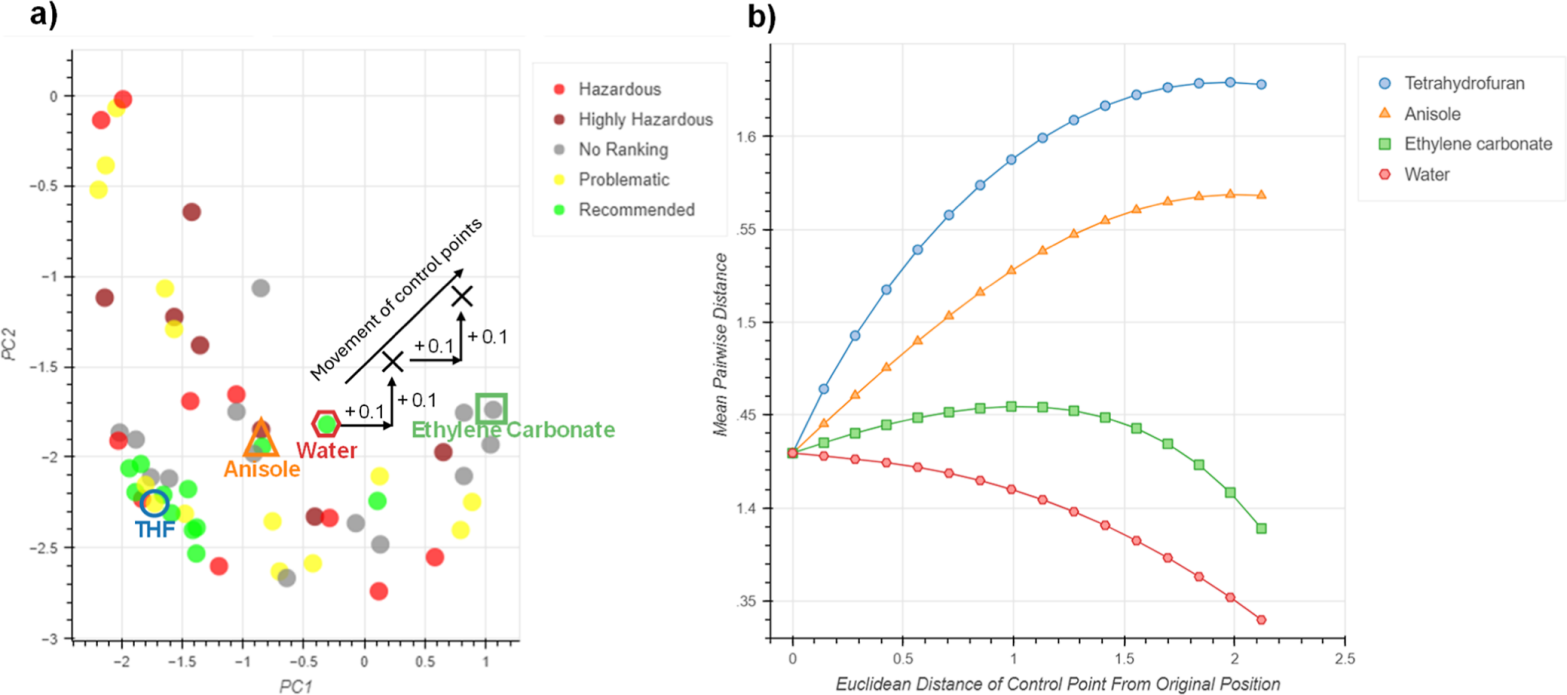
(a) Movement of control points emulating user interaction. Not to scale. (b) Effect of control point movement upon embedding distortion. Plot shows results for 15 iterations.

It was envisaged that an ideal embedding would maximize the spread of data points between the two principal components, making it easier to analyze trends and identify potential alternatives. A poorer embedding would instead place all data points close together and prove more difficult to analyze. The spread of data points can be measured by taking the mean of pairwise distances within an embedding, where larger values denote a larger average distance between data points and thus a more ideal embedding. Plotting this value vs the Euclidean distance traveled by a given control point for a range of movements gives a graphical representation of how the embedding is distorted by moving that control point (vide infra). For a solvent to be a good control point, its movement must lead to an increase in the mean pairwise distance. User actions were emulated by moving individual solvents through the 2D space in systematic increments (Figure 2a). The mean pairwise distance was calculated at each step and was plotted against the Euclidean distance moved by the control point from its original position (Figure 2b). Tetrahydrofuran, anisole, water and ethylene carbonate were chosen as the example control points to give a significant coverage of the embedding space. In all cases, the embedding distortion was small when the control points had moved a small distance from their original positions, while larger distortions were seen when they had moved further (Figure 2b).

The movements of ethylene carbonate and water result in an overall decrease of the mean pairwise distance as the control points move, signifying that the remaining solvents are moving closer together within the embedding. As this happens the structure of the data is lost, solvents begin to be grouped with other nonsimilar solvents and the potential to identify appropriate substitutions is diminished. Conversely, the movements of THF and anisole result in an overall increase of the mean pairwise distance. The notable difference between the four solvents is their proximity to other solvents in the initial embedding: THF has many close neighbors and its movement results in the most positive change, while water is almost entirely isolated, and its movement gives the most negative change.

**Figure 3:**
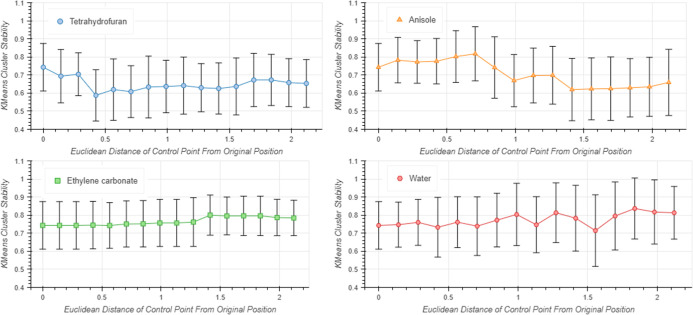
Cluster stability analysis for KMeans clustering at each movement step of a control point. Mean ARI values for 10 KMeans iterations are plotted, with standard deviation as error bars.

As an alternative method to assess the effect of moving a control point, cluster stability analysis was conducted on the PCA embedding at each movement step. The KMeans algorithm was used to find the clusters and was conducted 10 times for each embedding, varying the random seed parameter. The Adjusted Rand Index (ARI) was then calculated between each of the runs to measure the stability of the clusters, with the mean and standard deviation being plotted against the distance moved by the control point (Figure 3). The same control points that gave an increase to the mean pairwise distance (THF and anisole) also show a greater variability in the cluster stability compared to ethylene carbonate and water, which are much less variable. Despite this, the change in cluster stability is not statistically significant in any case.

**Figure 4:**
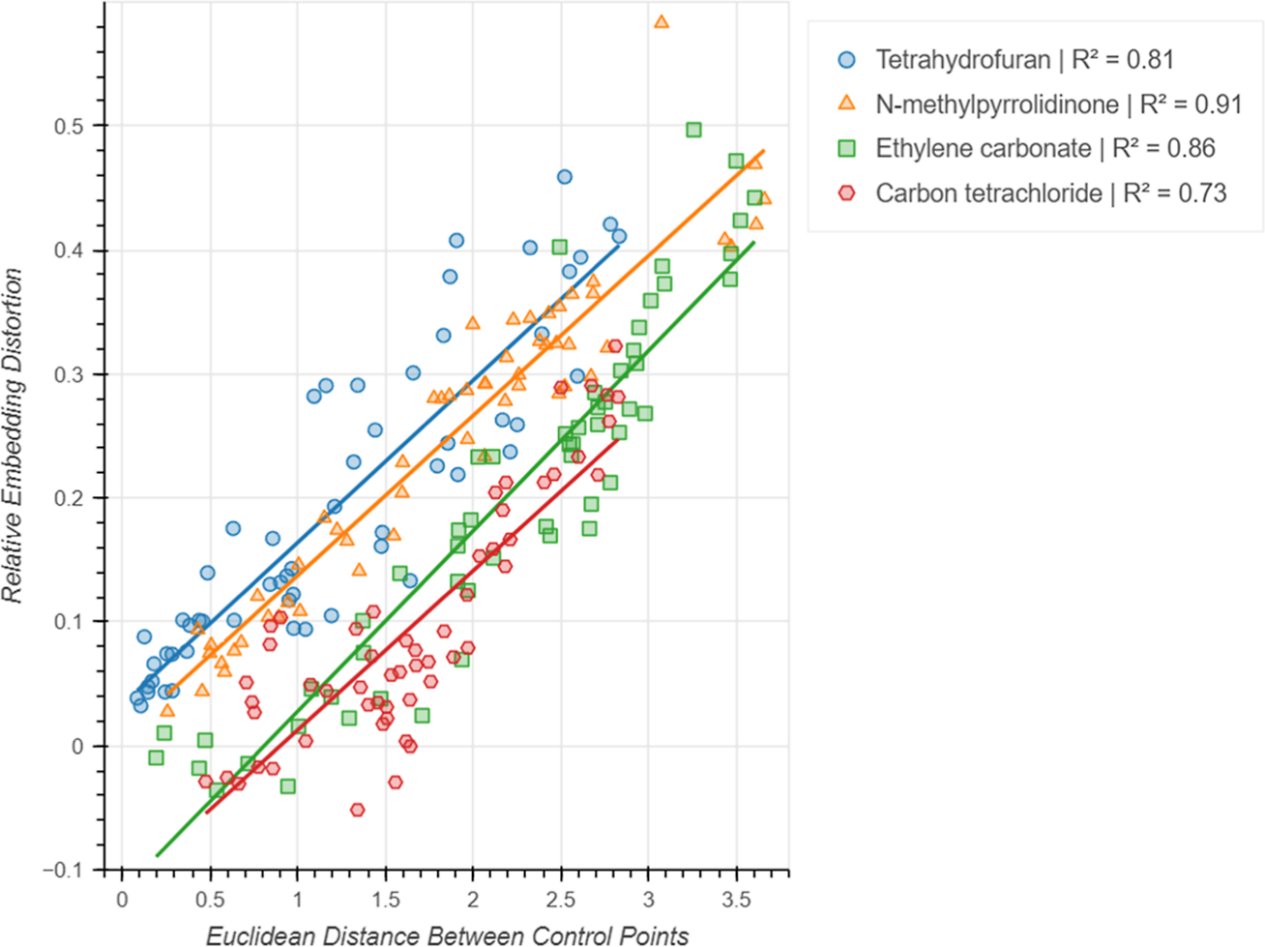
Linear trend between relative embedding distortion and control point distance for generated two-point embeddings.

Considering this information suggests that solvents with more neighbors would be ideal control points. However, this only reflects the movement of one point at a time. To emulate a true user interaction, the embedding distortion must be measured after grouping multiple solvents. To do this, THF was defined as a reference control point at its original position and each of the remaining solvents were sequentially moved toward it to provide the second control point. The embedding distortion was measured before and after the movement of the second point to calculate the relative distortion between the embeddings and this value was plotted against the initial Euclidean separation between the two control points (Figure 4). The results show a clear linear relationship between the relative distortion and control point separation, suggesting that it is more favorable to group solvents that are further away from each other in the initial embedding. Similar conclusions were reached using different solvents as the reference point, each showing the same general linear trend. This is a natural consequence of the embeddings being stable toward small movements of control points, as grouping solvents that are close together requires small movements which have little effect on the embedding distortion. As such, the distance between control points should be maximized when selecting them.

**Figure 5:**
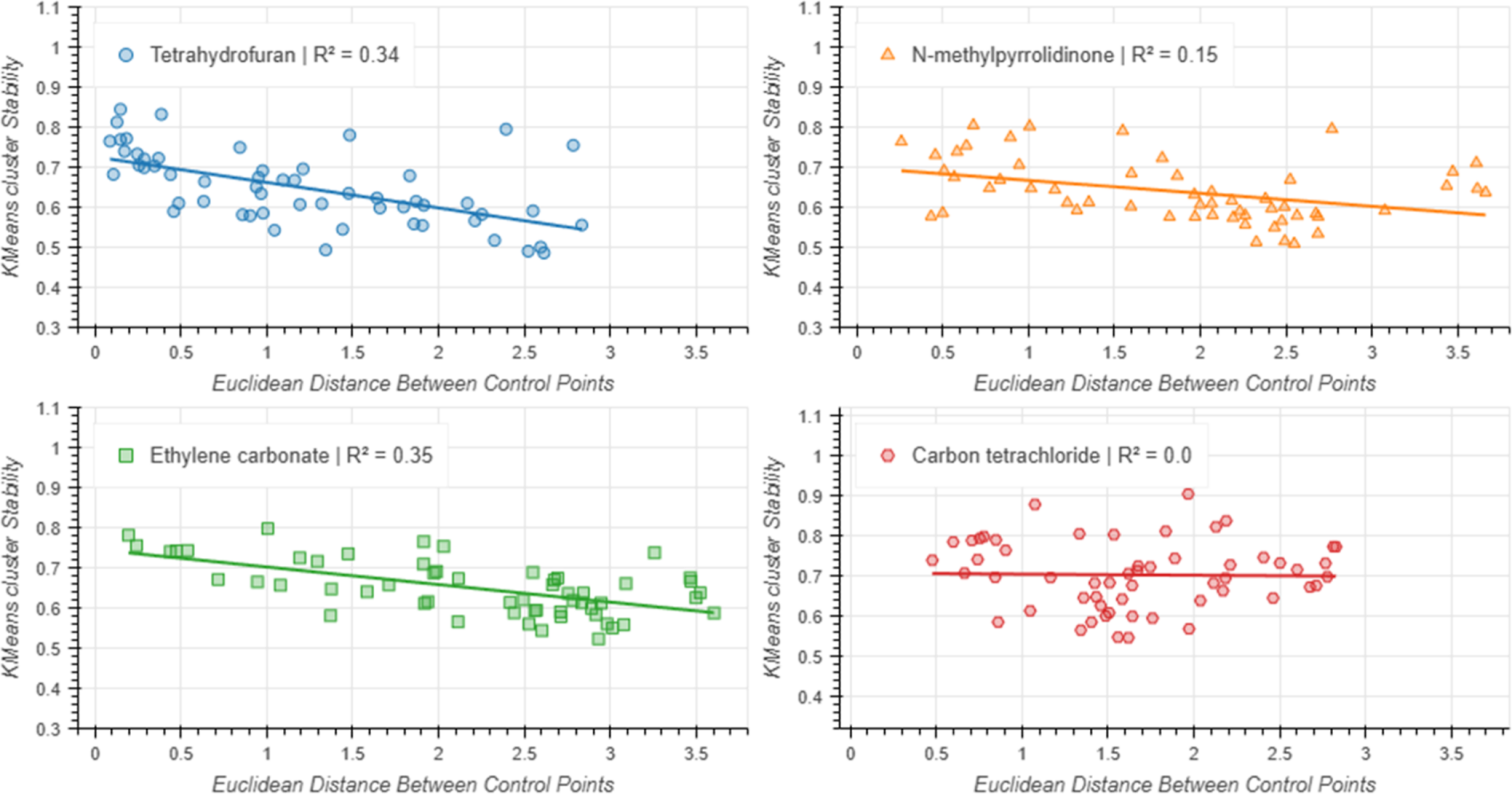
KMeans cluster stability vs control point distance for each generated two-point embedding. The mean ARI value is plotted. Error bars have been omitted for clarity, but these can be viewed in the Supporting Information.

As before, cluster stability analysis was conducted on each of the generated embeddings, and the mean values were plotted against the Euclidean distance between control points (Figure 5). These results showed that the cluster stability was weakly negatively correlated with the control point distance, apart from when carbon tetrachloride was used as the reference point. In all cases the change in cluster stability was statistically significant, which shows that the use of more than one control point has a significant effect on the embedding.

**Figure 6:**
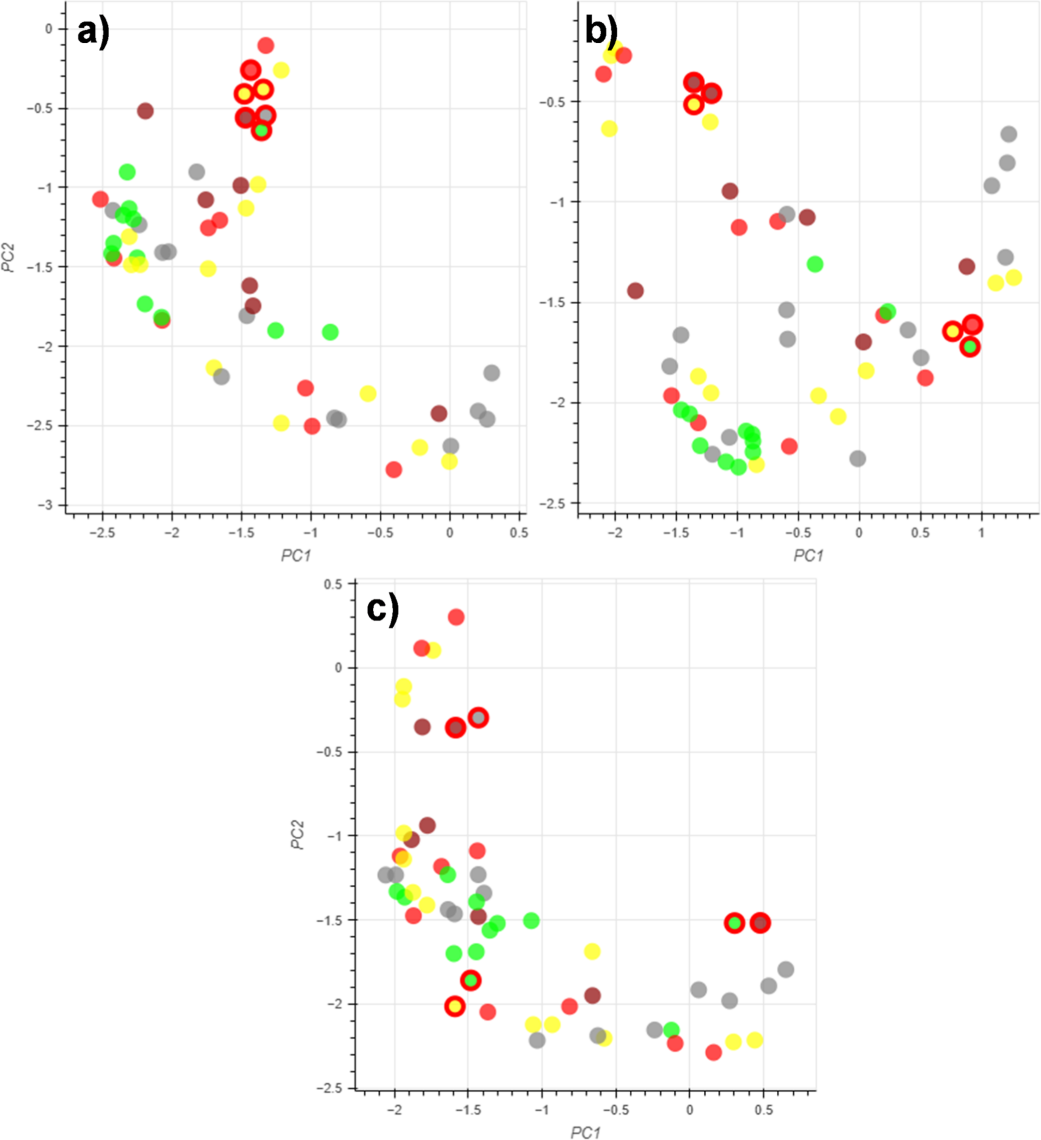
Examples of embedding with (a) one, (b) two and (c) three activity domains for six control points.

**Figure 7:**
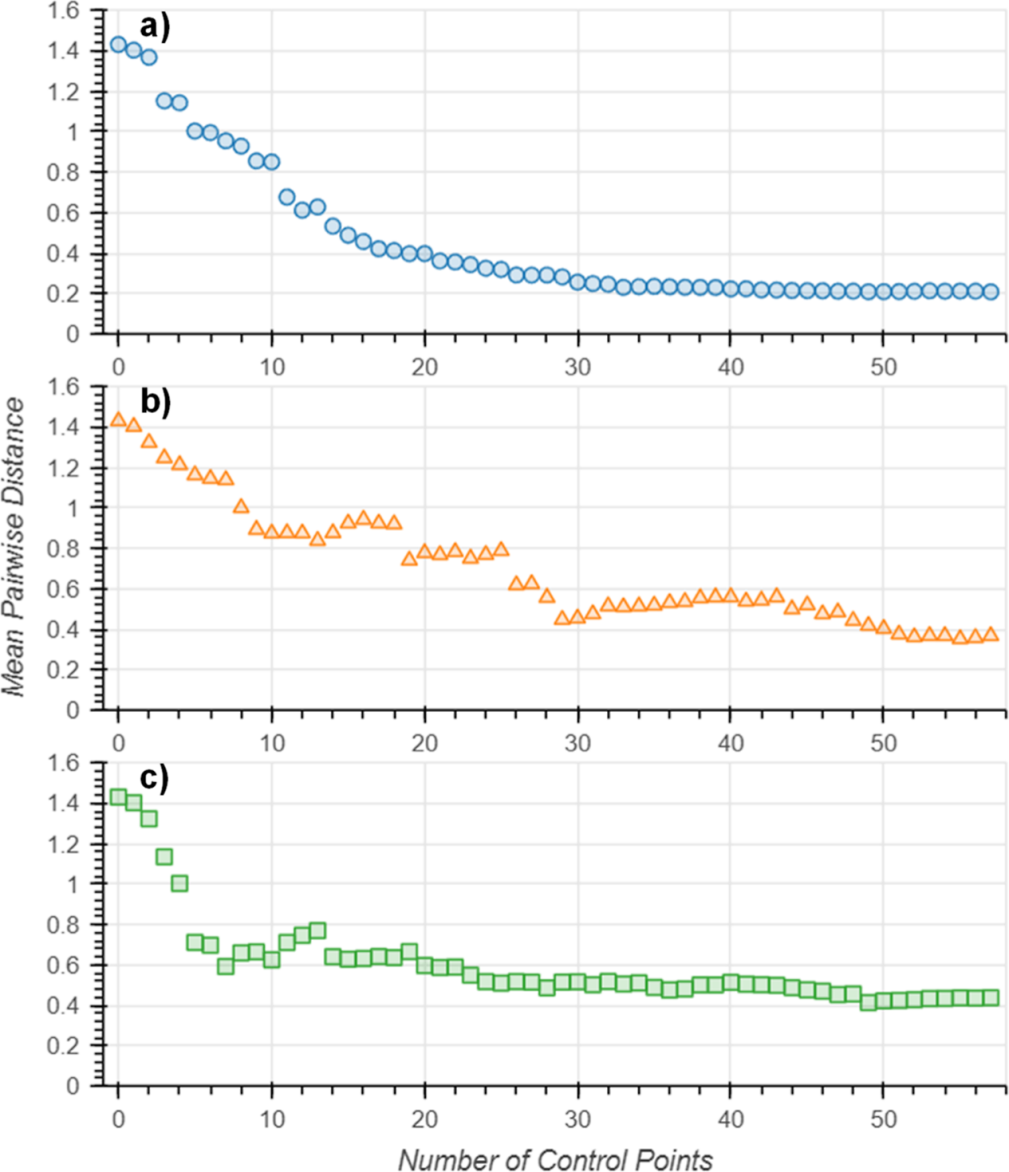
Embedding distortion versus number of control points for (a) one, (b) two and (c) three activity domains.

To identify the optimal number of control points for each activity domain, the embedding distortion was measured as a function of the number of control points (Figure 6). Initially solvents were grouped into one area of the embedding which showed a decay as more control points were added (Figure 7a). As real data is more likely to consist of multiple activity domains, solvents were then grouped into two and three areas of the 2D space to provide a more realistic example (Figure 7b,c, respectively). The two-domain example showed the steepest decline in the embedding distortion within the first ten control points, after which the gradient was shallower. A similar relationship was seen for the three-domain example, which also showed the steepest decline within the first ten control points, but the distortion was not affected by the addition of more control points. All three examples showed that grouping more than 30 control points lead to a plateau in the embedding distortion. These results suggest that the definition of any more than ten control points has little effect on the embedding distortion, and that no more than three to five control points should be defined per activity domain.

**Figure 8:**
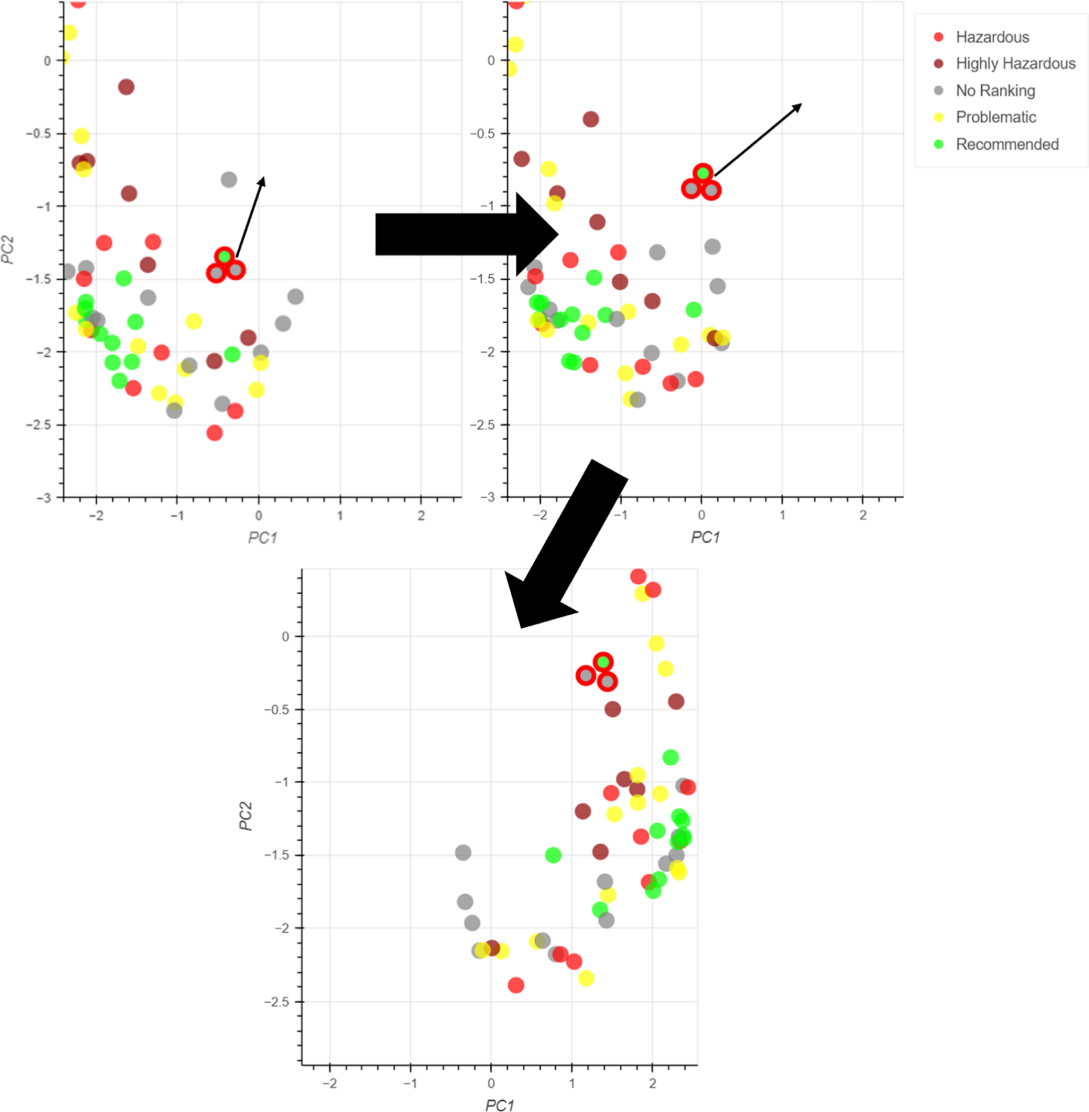
Movement of the same configuration of control points through the embedding. This was done for 100 iterations.

**Figure 9:**
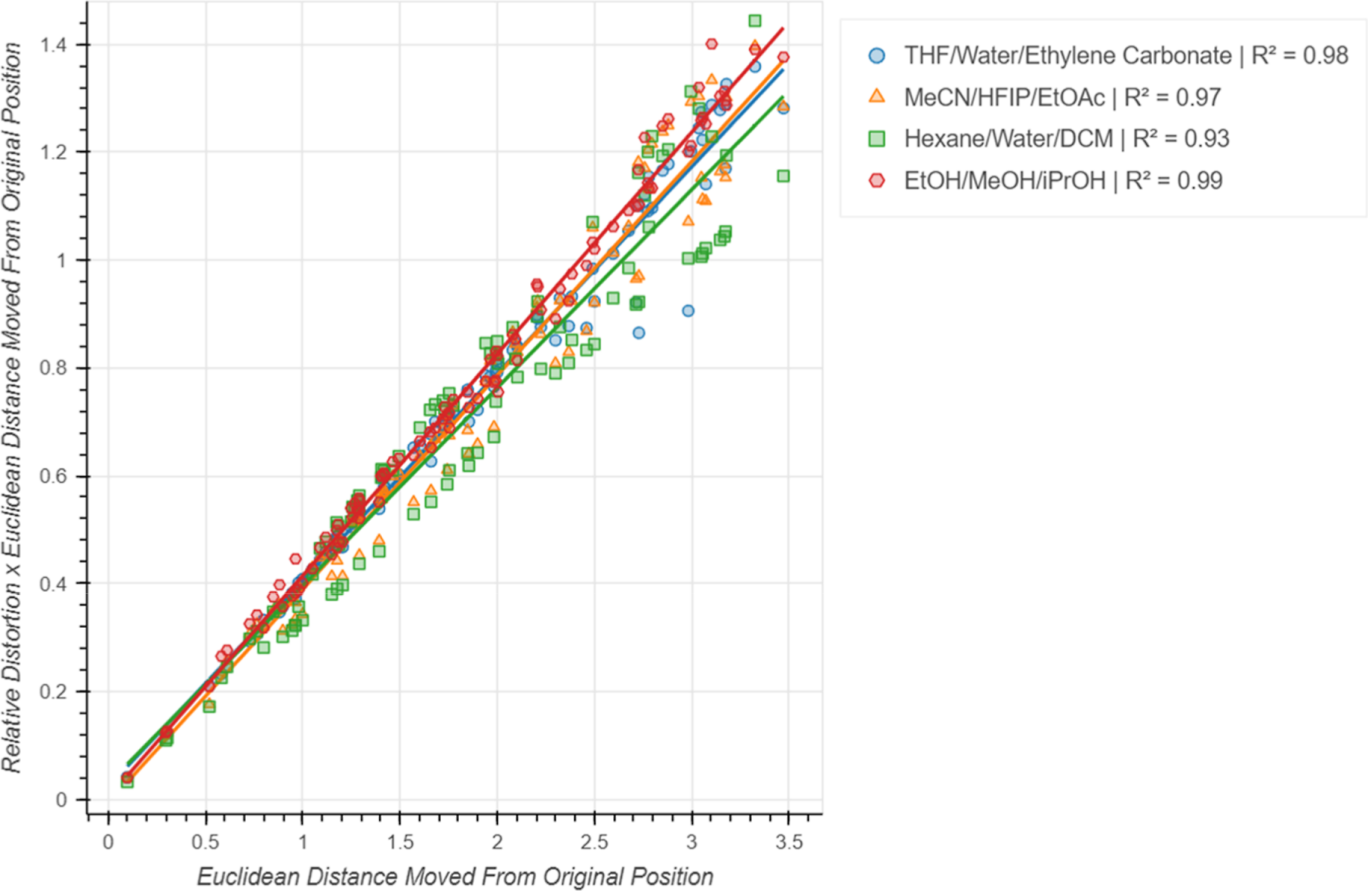
Embedding distortion multiplied by distance traveled vs distance traveled for constant configurations of control points. Values of embedding distortion were multiplied by the distance traveled to provide linear plots and is akin to multiplying by a constant value.

Finally, an investigation was undertaken to understand how the embedding responds when the same configuration of control points was placed at different positions in the 2D space (Figure 8). The relative positions of three solvents were defined and these were subsequently moved to 100 different positions within the bounds of the initial embedding, and the embedding distortion was measured after each movement. To ensure comparability across different embeddings, the distortion was defined as the mean pairwise distance divided by the maximum pairwise distance. The movement was measured as the Euclidean distance traveled by the first control points relative to its position in the initial embedding. Four groups of solvents were investigated, and in all cases the embedding distortion was shown to be almost constant with respect to the distance traveled. The plot shown in Figure 9 shows almost perfect linearity, with *R*^2^ values of above 0.90 in all cases. These results show that the embedding distortions are relatively stable with respect to the movement of the control point configuration, and that it is only the relative position of control points that affect the overall embedding.

**Figure 10:**
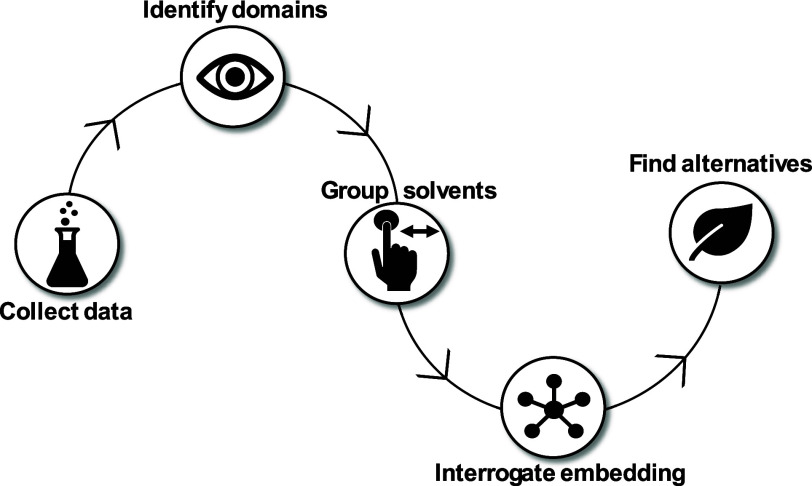
Schematic of evidence-based guidelines for interactive PCA.

With the considerations discussed above, the following guidelines were developed. These are simple to follow and encapsulate the results of this exploratory analysis. A visual schematic is shown in Figure 10:

(1) Identify activity domain from experimental data.
(2) Find solvents in diverse, populated areas.
(3) Group up to five solvents.
(4) Repeat steps (1) to (3) for other activity domains.
(5) Interrogate updated embedding and find alternatives.

### Case Studies

#### Thioesterification

**Figure 11:**
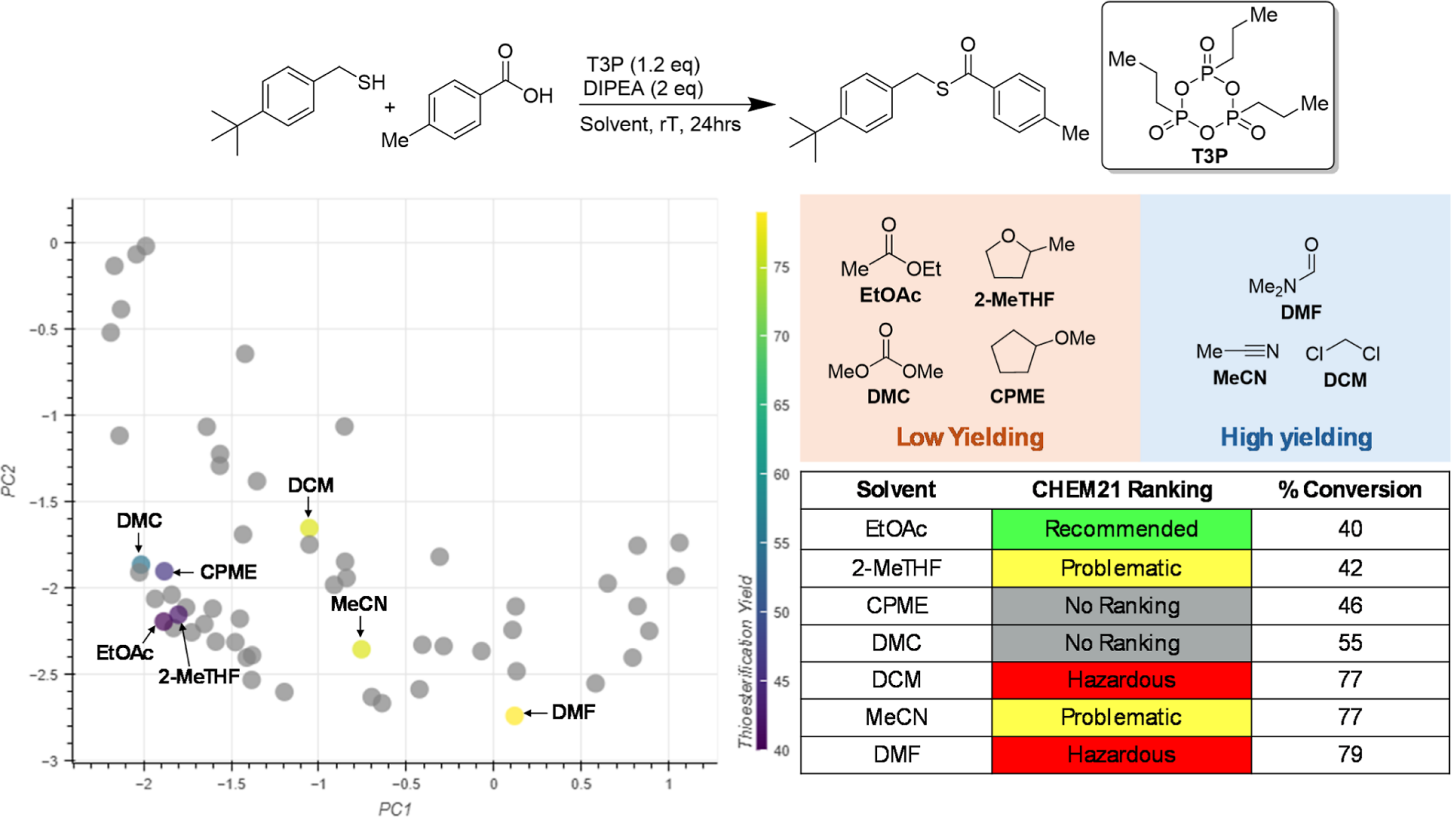
Thioesterification results chosen for experimental case study. Percentage conversions for each solvent, the corresponding activity domain and their position in the initial PCA embedding are shown.

To test the efficacy of these developed guidelines, the interactive PCA was applied in the context of synthetic data from the literature. A study by Jordan and Sneddon,^36^ which investigated the effects of solvents on thioesterification reactions (Figure 11), was chosen for this purpose as the range of solvents examined gave a significant coverage of the PCA embedding. According to the guidelines, the experimental data were used to identify the activity domains (Figure 11). The first of these contained four low yielding solvents that gave less than 55% conversion: dimethyl carbonate (DMC), ethyl acetate (EtOAc), cyclopentyl methyl ether (CPME) and 2-methyl-tetrahydrofuran (2-MeTHF). Only EtOAc was labeled as “Recommended” according to the CHEM21 selection guide. The second activity domain contained the higher yielding solvents: acetonitrile (MeCN), dichloromethane (DCM) and dimethyl formamide (DMF), none of which have a lower CHEM21 ranking than “Problematic”. Identifying these on the initial embedding shows that the low yielding solvents are clustered close together, making it likely that these would be placed together in any updated embeddings (Figure 11). The high yielding solvents, however, were more spread out, which would provide a more rigorous test for the interactive PCA.

With the activity domains identified, attention turned to identifying solvents in diverse, populated areas. For the low yielding solvents, it was evident that the structure of the domain was reflected in the initial embedding. EtOAc and 2-MeTHF both gave ∼40% conversion and are grouped together in a slightly separate region to DMC and CPME, which gave higher conversions of 46 to 55%. Though the EtOAc/2-MeTHF cluster is more populated, the guidelines clearly state control points should also be chosen from diverse areas. Therefore, EtOAc and DMC were chosen as the low yielding control points. For the higher yielding domain, selecting control points was more difficult as neither DMF nor MeCN are located within populated areas. However, identifying the highest yielding solvent (DMF) without using it as a control point would provide a good demonstration of how the interactive PCA could be used to guide solvent screens. Consequently, MeCN and DCM were selected as the control points for the high yielding domain. The remaining solvents provide an opportunity to assess the updated embeddings relative to experiment.

**Figure 12:**
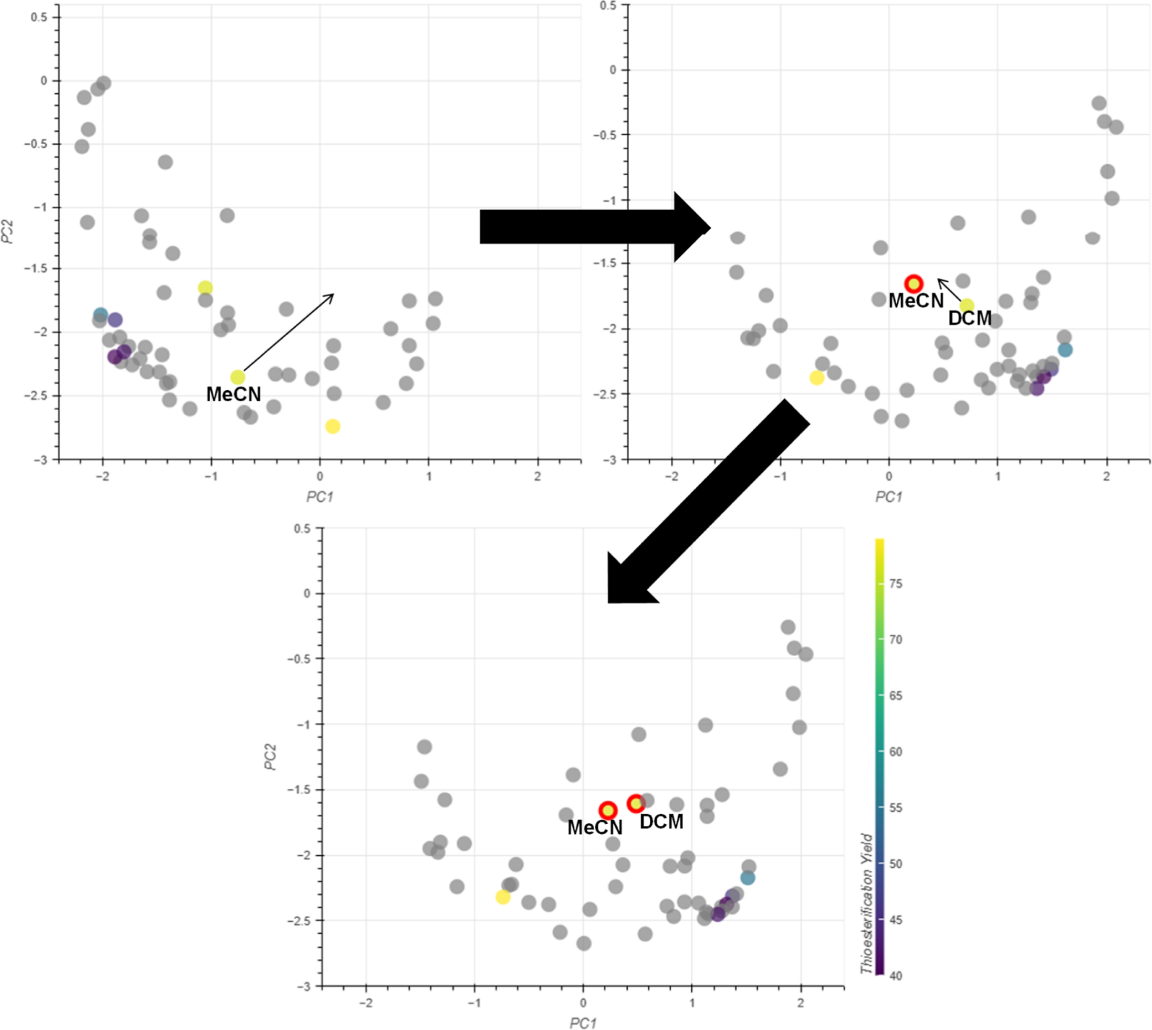
Defining the high-yielding domain using experimental data.

**Figure 13:**
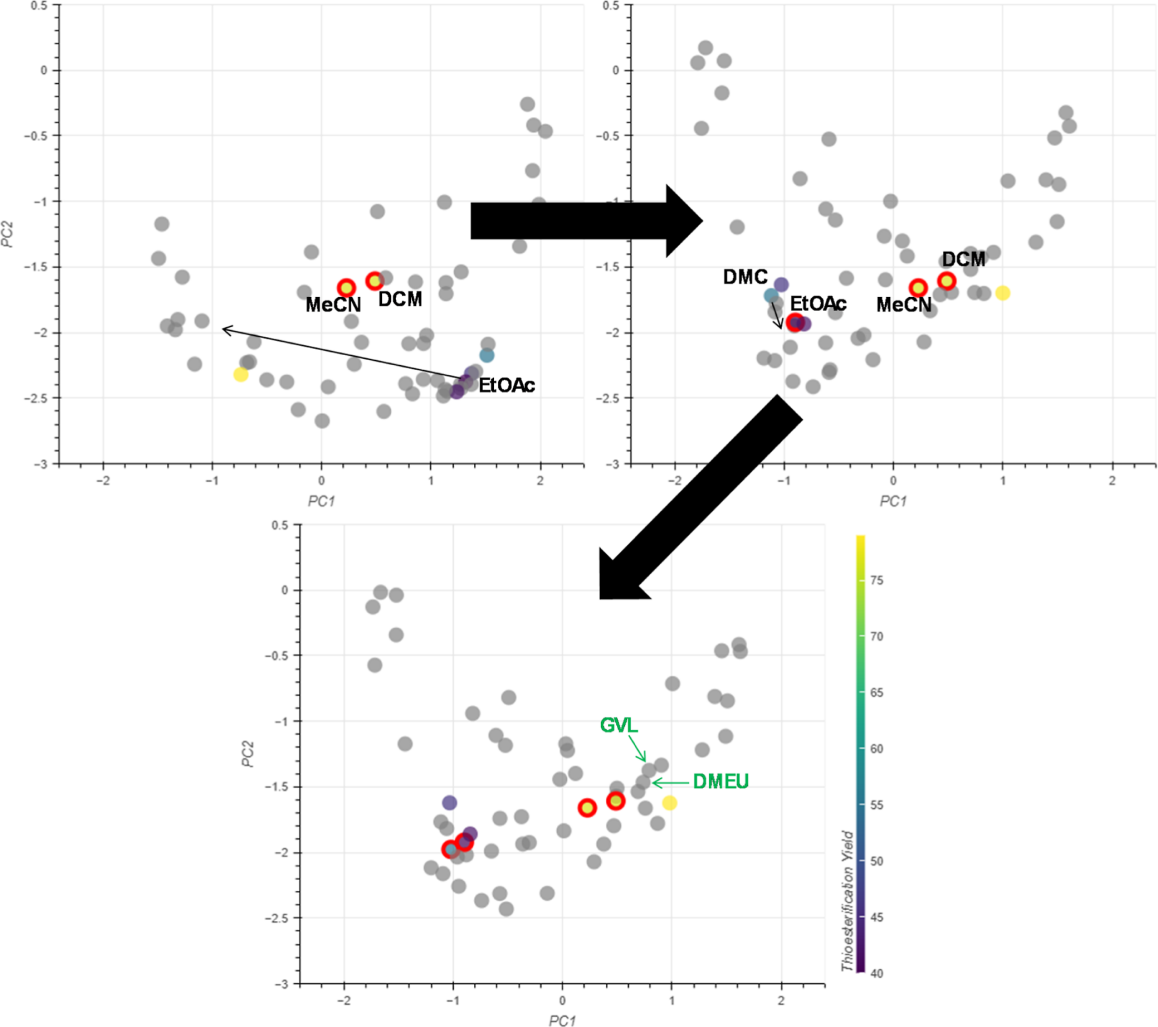
Grouping low yielding solvents using experimental data. Solvents GVL and DMEU are highlighted in green.

Next, the chosen control points were grouped. DCM and MeCN were brought together to define the high yielding domain in the updated embedding (Figure 12). DMF was not clustered within this domain showing that this embedding did not match the experimental data. Defining EtOAc as a control point further away from the high yielding solvents led to a significant improvement, with both sets of solvents being appropriately grouped (Figure 13). This three-point embedding shows good agreement to experiment with all high yielding solvents being grouped together and provides a platform for the identification of potential solvent substitutions. The greener solvents close to the high yielding domain in Figure 13 correspond to ethylene glycol and water. γ-Valerolactone (GVL) and dimethylethylene urea (DMEU) are also included in this cluster, and while these solvents were not assessed as part of the CHEM21 selection guide they are considered as sustainable solvent alternatives.^37^ Their position in the embedding suggests that any of these solvents may provide similarly high yields for this reaction (Figure 13).

The previously discussed structure of the low-yielding domain is also maintained in this embedding, which further confirms the validity of the embedding according to experimental data. As may be expected, this structure is lost if DMC is defined as a control point next to EtOAc. This change only alters the position of DMC, while those of 2-MeTHF and CPME remain unaffected. A larger effect is seen within the high yielding domain with DMF moving closer to the MeCN and DCM control points, though this offers little more information. The same solvents can be identified as alternatives as in the previous embedding, suggesting that the addition of this fourth control point is not needed.

This case study demonstrates a practical example of how interactive knowledge-based kernel PCA can be used to identify solvent alternatives. Using experimental data from just three solvents to update the embedding, the highest yielding solvent and several alternatives could be identified. The same is true for poorly performing solvents, providing a truly tailored guide for solvent substitution and selection.

#### Reductive Amination

**Figure 14:**
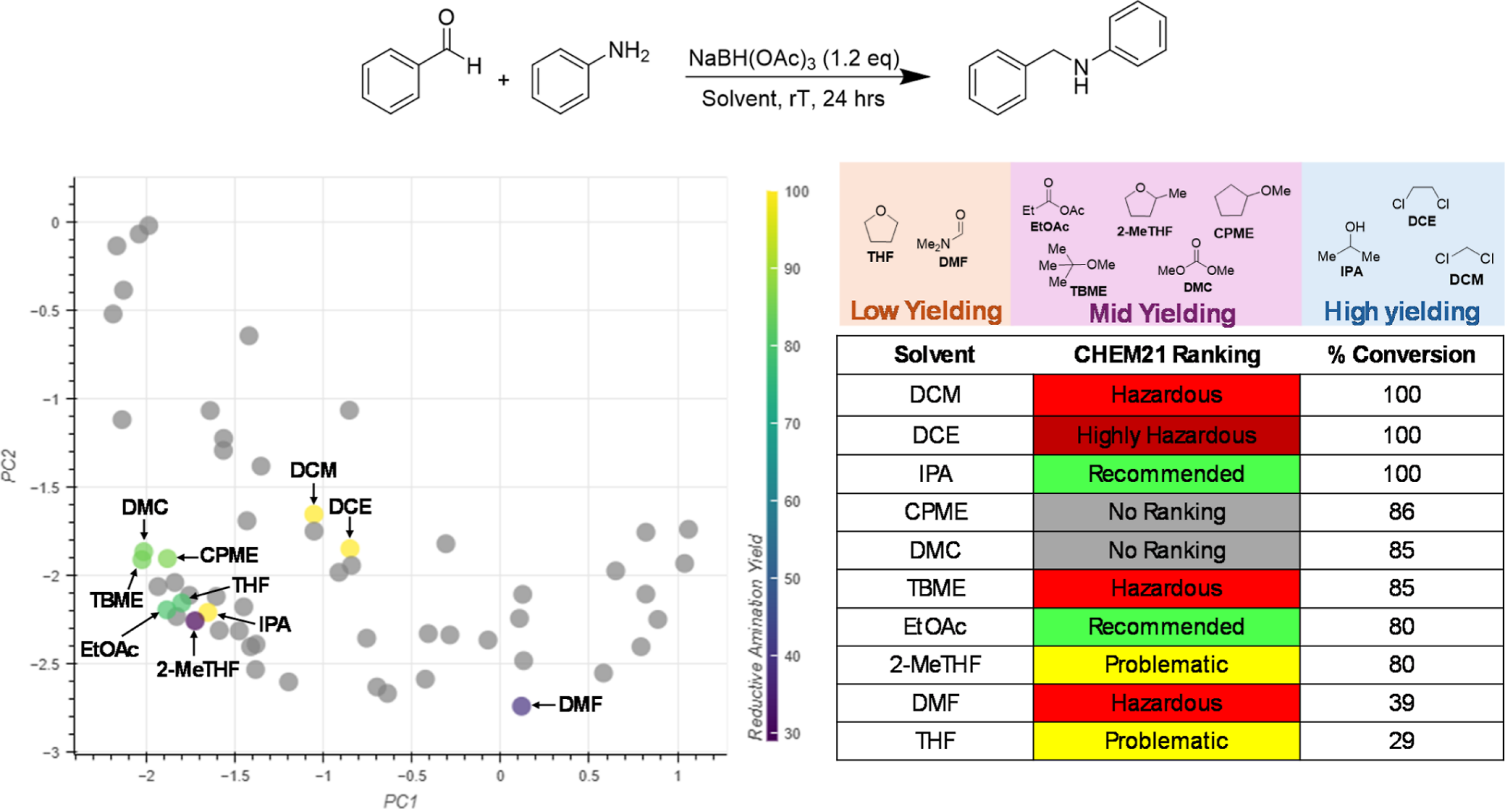
Experimental data for the reductive amination of benzaldehyde with aniline.^38^

Another application was within the context of the reductive amination of benzaldehyde with aniline (Figure 14). These data were taken from a study by McGonagle et al.^38^ which aimed to develop a solvent selection guide for a range of reductive amination reactions. The solvents included in this study can be split into three activity domains as low yielding (<40% conversion), mid yielding (80–90% conversion) and high yielding (100% conversion), and their positions in the initial embedding can be seen in Figure 14. Though most of the solvents are grouped close together in this embedding, the structure of the yield data is not reflected. The solvents IPA, THF and 2-MeTHF are located close together, but these all belong to different activity domains. Using interactive PCA, it is possible to reflect this experimental data in the embedding.

**Figure 15:**
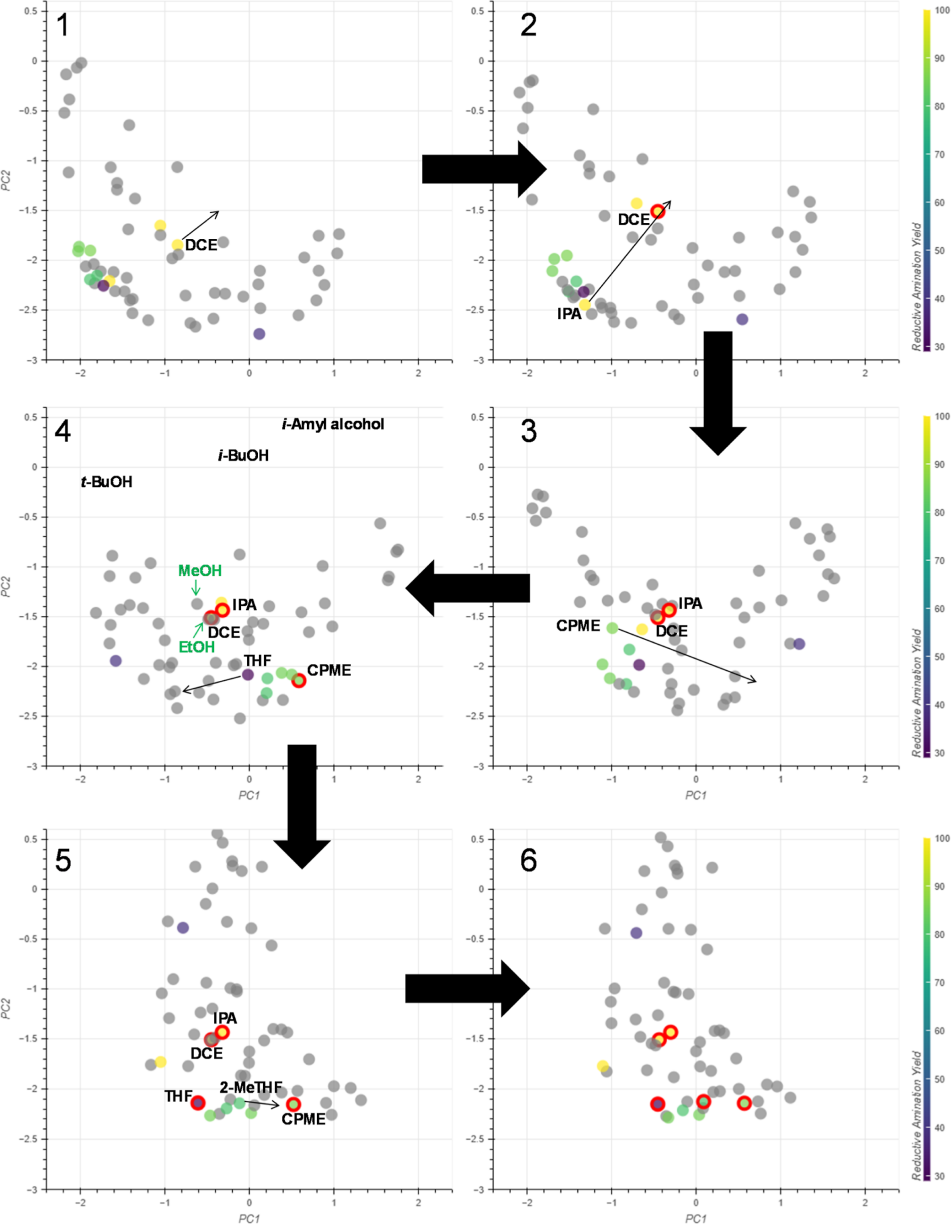
Movement of control points relative to reductive amination data.

For the high yielding domain, DCE and IPA were grouped together (Figure 15, embeddings 1–3), which placed DCM close to the high-yielding solvents (Figure 15, embedding 3). However, CPME was also placed close to the high yielding solvents which was problematic, as it belongs to the mid yielding domain. Moving CPME away from the high yielding domain (Figure 15, embedding 3–4) successfully groups the other mid yielding solvents: DMC, TBME, EtOAc and 2-MeTHF nearby CPME. The low yielding solvent DMF is also placed far from the other solvents in this updated embedding. However, 2-MeTHF is grouped with the other mid yielding solvents despite being a low yielding solvent. Simply defining 2-MeTHF as a control point further from the mid yielding solvents causes them to be spread between the 2-MeTHF and CPME control points, leading to a less successful grouping (Figure 15, embedding 5). Attempts to define 2-MeTHF as a control point close to CPME were unsuccessful (Figure 15, embedding 6). This had little effect on the overall embedding and reached a maximum distance from the 2-MeTHF control point that could not be increased.

Embeddings 5 and 6 do not match the experimental data, as the position of DCM is placed further away from the other high yielding solvents. The embedding that best captures the experimental data (excluding 2-MeTHF) is embedding 4 and the potential substitutions are alcohols: methanol, ethanol, *t*-butanol, *i*-butanol and *i*-amyl alcohol.

#### Solubility

The interactive PCA was also evaluated in the context of drug solubility for a range of organic solvents. Such an application could serve to highlight more sustainable solvents for dissolving drug-type compounds while simultaneously identifying more sustainable antisolvents for crystallizations. The data used for this case study were taken from a recent publication that aimed to predict the solubility of organic compounds using machine learning.^39^ The data are publicly available on GitHub at: https://github.com/AntonyVass/cmac_solpred_cosmo_rf and the data for the solubility of paracetamol were taken from https://github.com/AntonyVass/cmac_solpred_cosmo_rf/blob/main/dataset_minus_confidential.txt.

**Figure 16:**
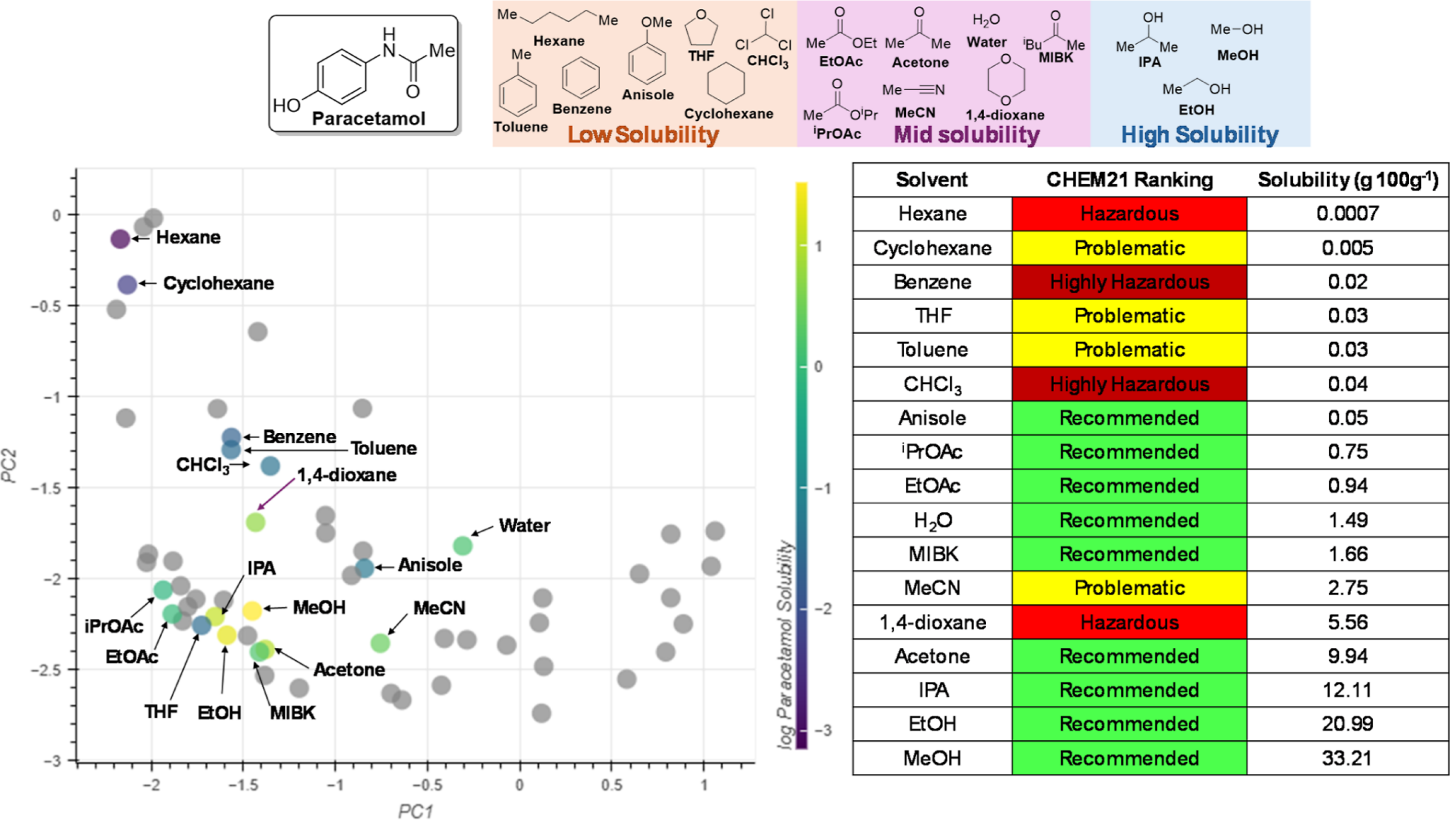
Solubility data for paracetamol in a range of organic solvents.

The solubility of paracetamol in a range of solvents is shown in Figure 16 and is measured as the grams of solute per 100 g of solvent (g 100 g^–1^). From these, three activity domains were identified corresponding to low solubility (<0.1 g 100 g^–1^), mid solubility (<10 g 100 g^–1^) and high solubility (>10 g 100 g^–1^).

**Figure 17:**
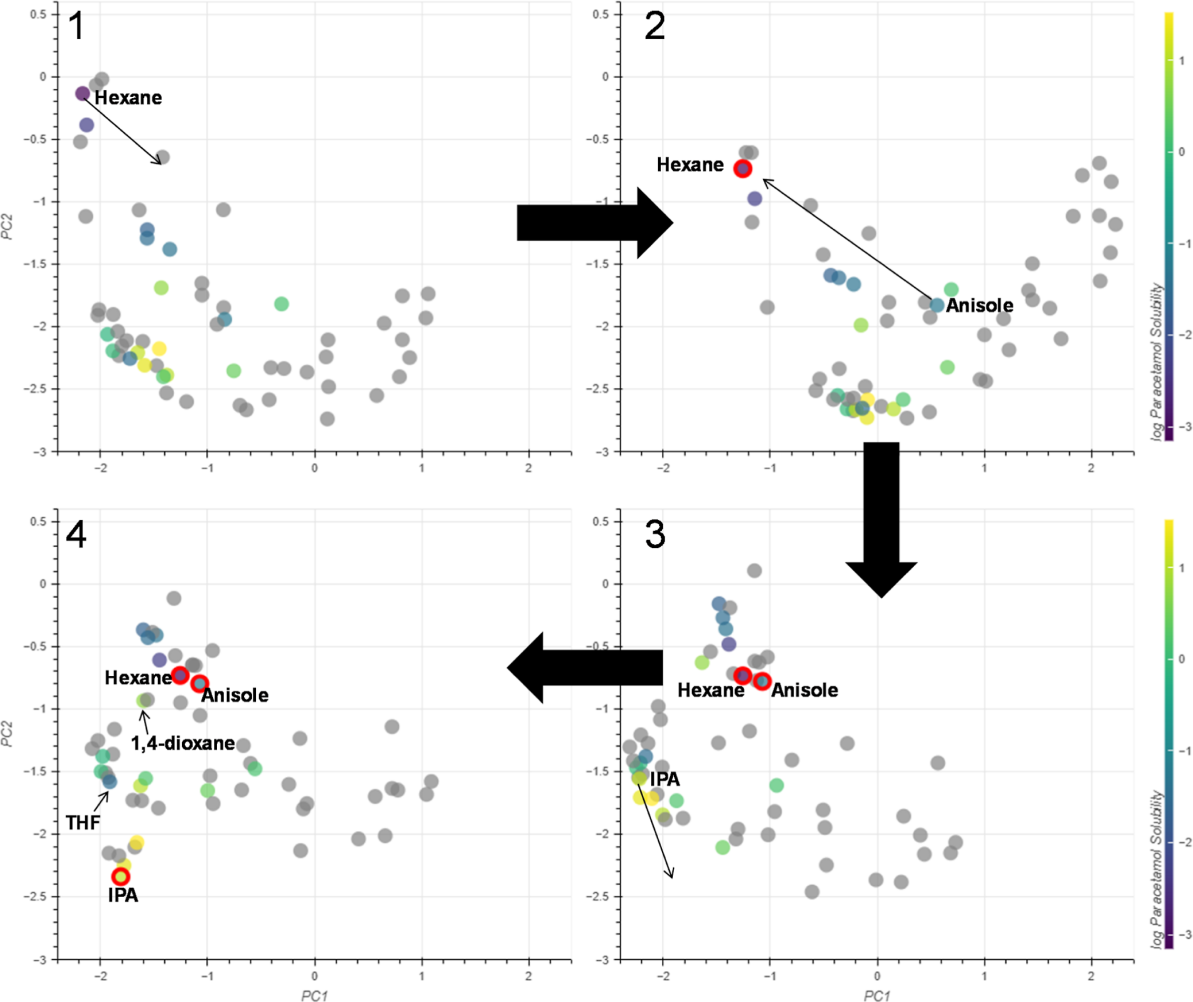
Movement of control points relative to paracetamol solubility data.

In the low solubility domain, hexane and anisole are the furthest solvents apart and so were chosen as control points. Grouping these together produced an embedding that grouped nearly all solvents that gave low solubility (Figure 17, embedding 3). However, the positions of THF and 1,4-dioxane did not match the experimental results. Using IPA as a control point for the high solubility gave an embedding that was much closer to the solubility data (Figure 17, embedding 4), clustering the remaining high solubility solvents (EtOH and MeOH). This is unsurprising as all solvents within the high solubility domain are alcohols and are predisposed to be grouped with other alcohols. As such, using an alcohol as a control point would naturally group the alcohols together. Despite this, embedding 4 also captures the information of the mid solubility domain, with solvents such as EtOAC, ^*i*^PrOAc and MIBK being located at the midpoint between the low and high solubility domains.

The position of 1,4-dioxane in embedding 4 is ambiguous, as it is found at the interface between the low and mid solubility domains. Experimentally, 1,4-dioxane belongs in the mid solubility domain, but its position in embedding 4 would suggest more similar behavior to anisole rather than acetone. The guidelines state that using 1,4-dioxane as a control point would have little effect on the embedding as it has few neighbors, and this was observed to be the case (see Supporting Information for details).

The position of THF is less ambiguous, being located firmly in the mid solubility domain. This does not match the experimental data, as THF was identified as being a low solubility solvent but embedding 4 suggests that THF should belong to the mid solubility domain. Moving THF to the low solubility domain as a control point alters the positions of all other solvents, which removes all agreement to the experimental data (see Supporting Information for details).

The remaining points in embedding 4 show good agreement to the experimental data and the three solubility domains are well-defined. As the solvents that give the highest solubilities are all classified as “Recommended” by the CHEM21 selection guide,^16^ no greener alternatives can be identified. Though the low solubility domain contains almost no “Recommended” solvents, switching from a “Hazardous” solvent to a “Problematic” solvent is a step in the right direction.

Despite this, the identification of solvent alternatives may still be useful for users that are interested in different criteria such as cost, physical solvent properties, or compatibility with previous/subsequent processes.

#### Limitations

**Figure 18:**
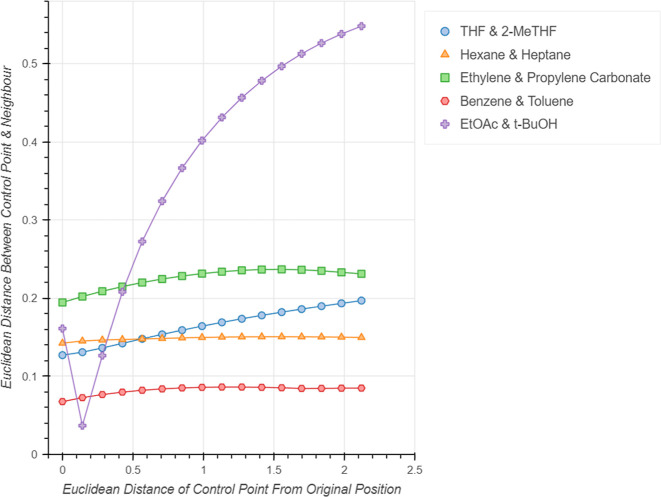
Visualization of the activity cliff limitation.

A limitation of interactive PCA for solvent selection is the inability to account for “activity cliffs”.^40^ In drug discovery, activity cliffs are defined as large differences in drug potency that can be attributed to small changes in molecular structure. This can also be observed during solvent screening, with solvents such as THF and 2-MeTHF giving drastically different reaction yields despite their close structural similarity.^38,41^ The interactive PCA however cannot account for such effects, as the input solvent descriptors are based solely on physicochemical properties and do not include any steric information. As such, these two solvents are always placed in proximity within any calculated embedding, suggesting that they are interchangeable. As THF is moved through the embedding the distance between the two solvents remains consistent, especially relative to the largest distance between solvents in the embedding (Figure 18). This effect is also seen for other structurally similar solvents, such as hexane/pentane, ethylene carbonate/propylene carbonate and benzene/toluene. For reference, the distance between two unrelated solvents, EtOAc/*t*-butanol, was also measured as EtOAc was moved through the embedding and plotted in Figure 18. This shows the expected behavior, with the distance increasing as the control point is moved. Users should be aware of this limitation.

#### Integration into AI4Green

**Figure 19:**
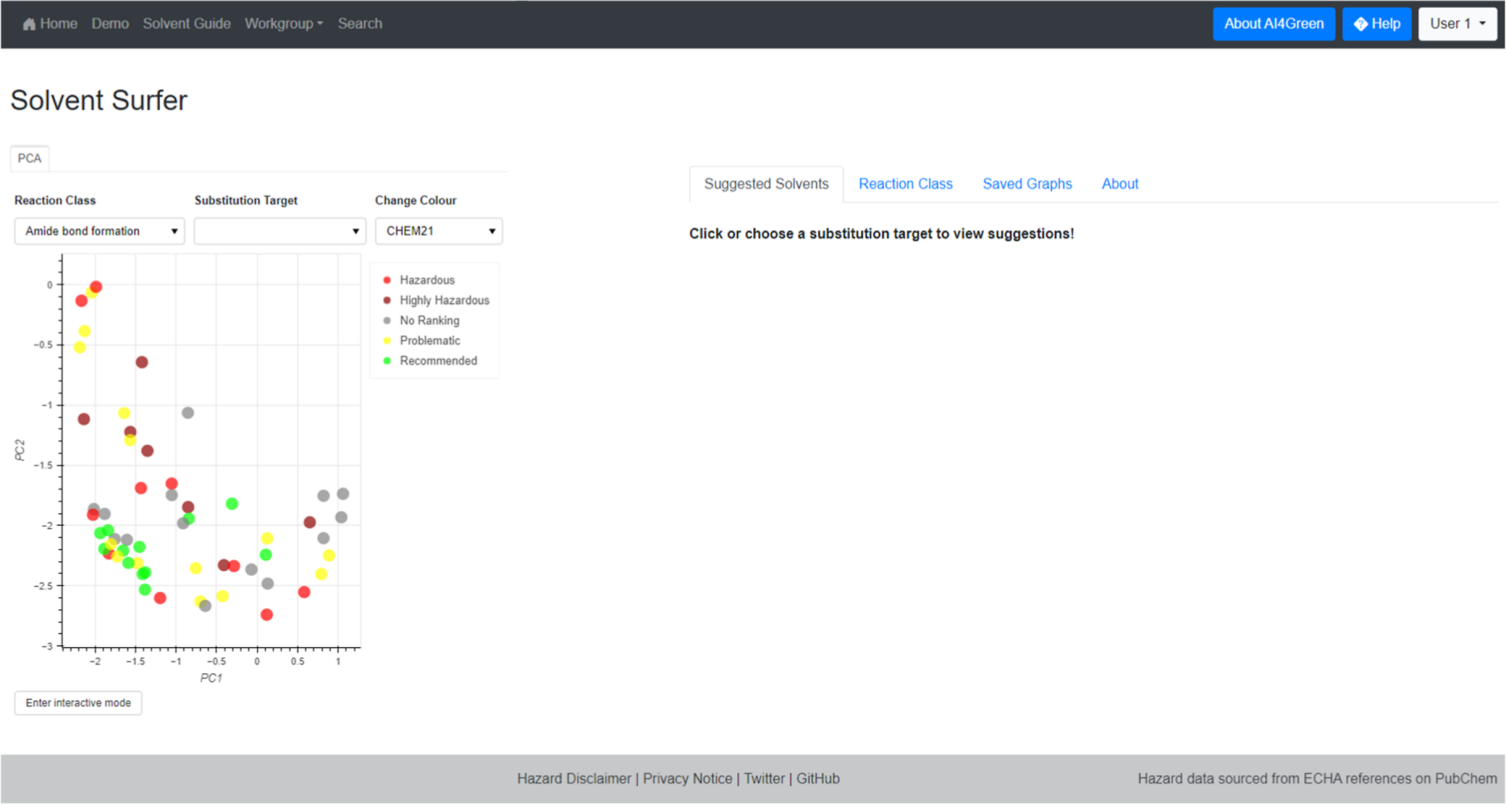
Integration of solvent PCA into AI4Green.

To make this a useful tool for synthetic chemists, it must be easily accessible and easy to use. To achieve this, the interactive knowledge-based kernel PCA has been integrated into AI4Green as the latest sustainability plugin, the Solvent Surfer. It can be accessed within AI4Green and is split into two sections containing the PCA plots on the left and informational tabs on the right (Figure 19). Selecting a reaction class from the “Reaction Class” dropdown menu generates a static, reaction-specific kernel PCA embedding, using a tailored descriptor set that reflects only the most important solvent parameters. This is a form of explainable AI, allowing users to understand precisely which descriptors are being used to generate the embedding. Some reaction classes are accompanied by experimental data that can be visualized by selecting the corresponding color from this same dropdown. Information and references for each reaction class are provided under the “Reaction Class” tab, which describes how the descriptors for each class were chosen and gives a summary of the experimental data included in the visualizations.

The data points are colored by default according to the CHEM21 ranking system to provide an easy visualization of solvent “greenness”, but this can be changed to reflect any of the descriptors by selecting a color in the “Change Colour” dropdown menu.

**Figure 20:**
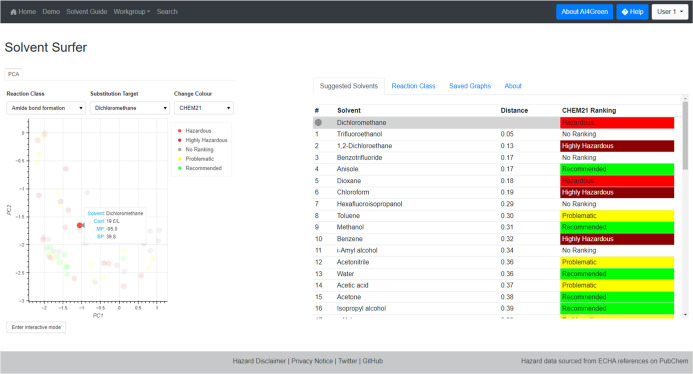
Generation of Suggested Solvent table by selecting a solvent from the Substitution Target dropdown menu. The summary for Dichloromethane is shown when the solvent is hovered over, and clicking the data point also generates the suggested solvent table.

Selecting a solvent from the “Substitution Target” dropdown highlights the corresponding point on the plot and displays a table that summarizes the Euclidean distances between the chosen and all other solvents. This table is generated under the “Suggested Solvents” tab and can be used to identify suitable alternatives, highlighting which solvents are closest and providing the CHEM21 rankings alongside (Figure 20). Hovering over a solvent within the plot displays its name, cost, melting point, and boiling point while clicking on a solvent generates the same distance table as selecting a value in the “Substitution Target” dropdown.

**Figure 21:**
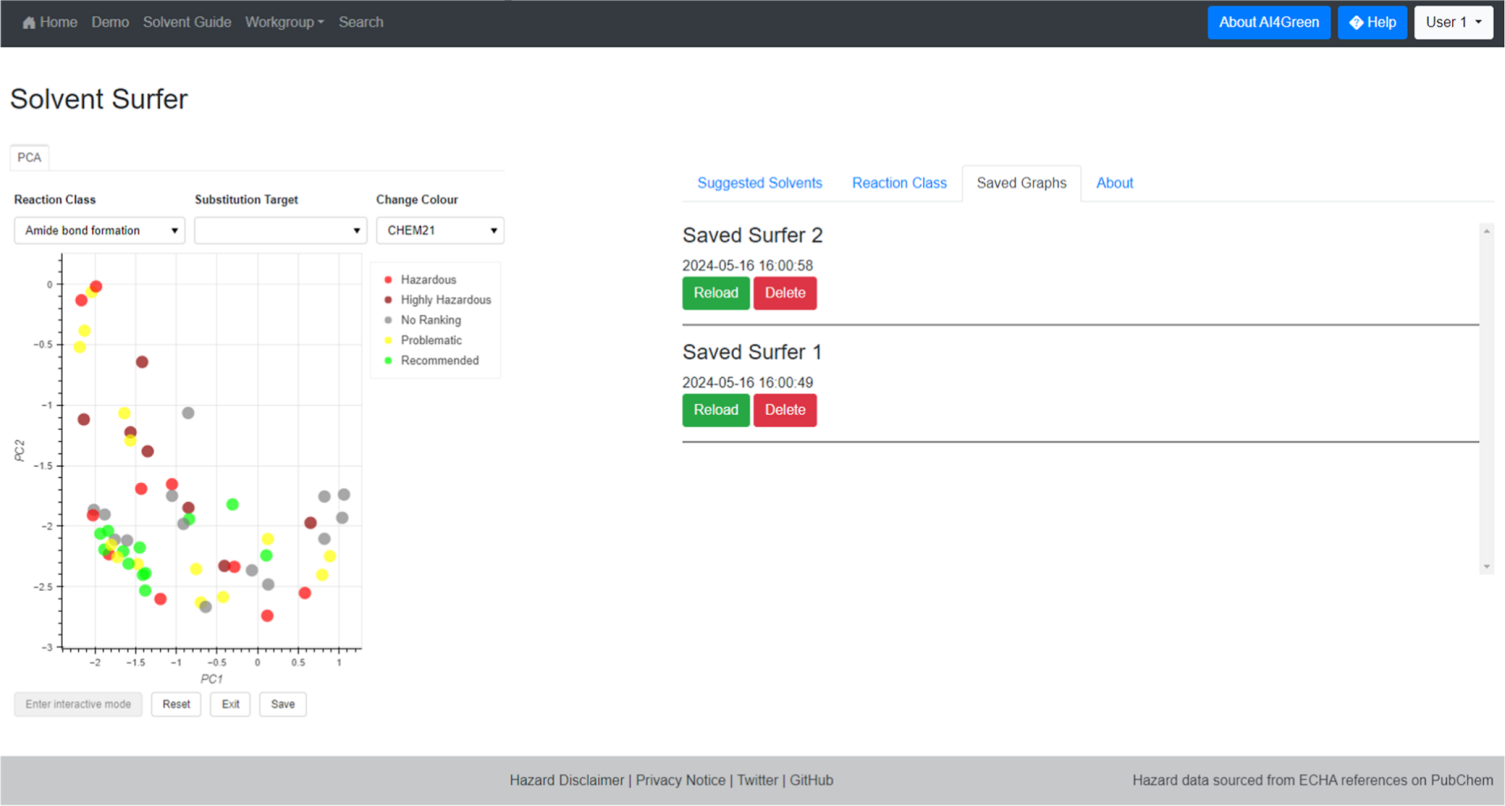
Solvent Surfer in interactive mode with content of “Saved Graphs” tab shown.

**Figure 22:**
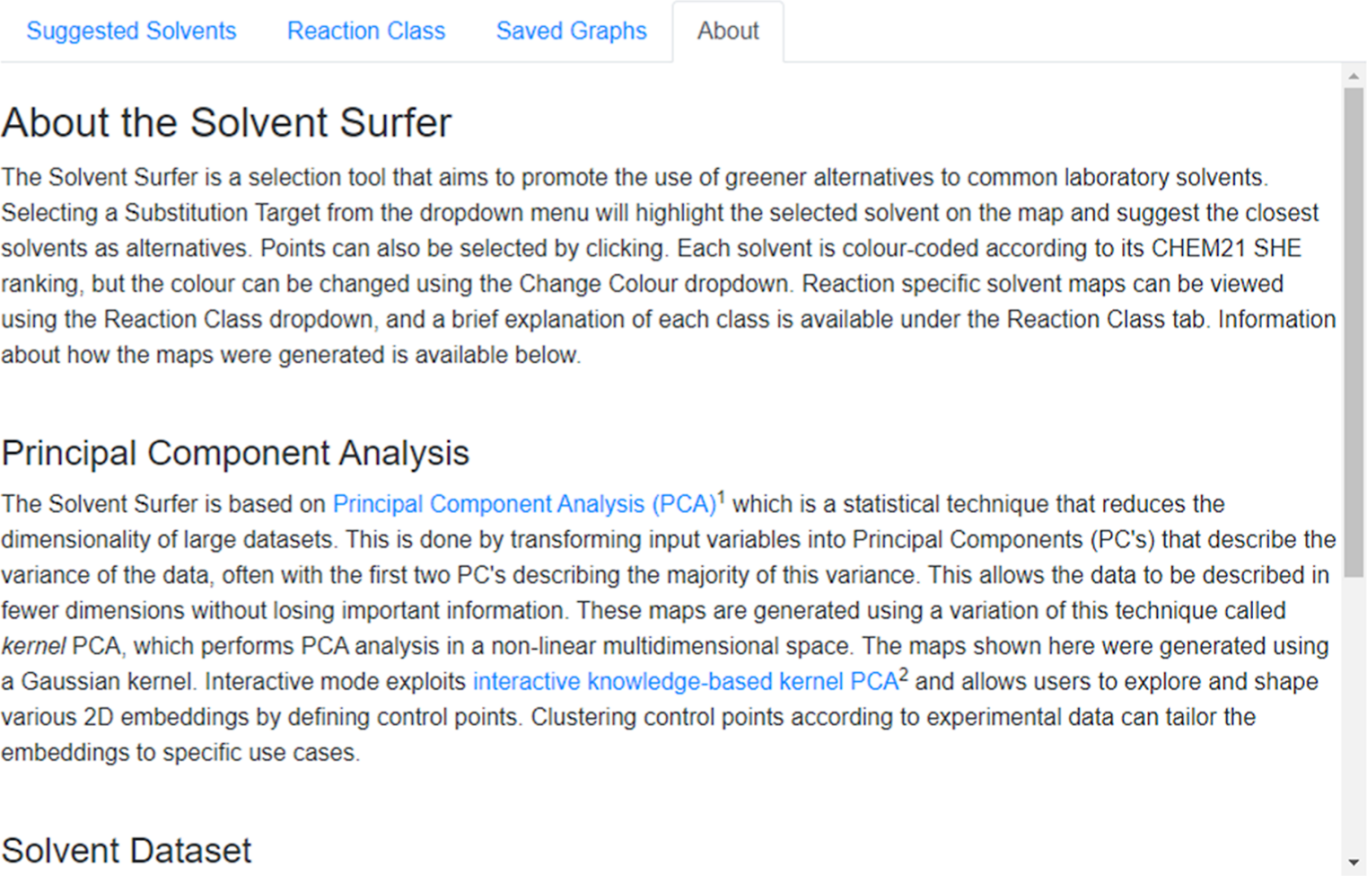
Content of the “About” tab adjacent to the Solvent Surfer.

Interactive PCA can be initialized by clicking on the “Interactive Mode” button, which allows users to begin clicking and dragging solvents. The embedding is automatically updated when the solvent is dropped, and the control point is highlighted with a red outline. Updated embeddings can be saved to the user profile and reloaded from under the “Saved Graphs” tab, and all changes can be reversed by clicking the “Reset” button which returns the embedding to its initial state (Figure 21). Exiting interactive mode allows users to interrogate updated embeddings as before, by changing colors or selecting solvents as substitution targets. The “About” tab provides some general information about PCA, interactive mode, and solvent selection (Figure 22).

**Figure 23:**
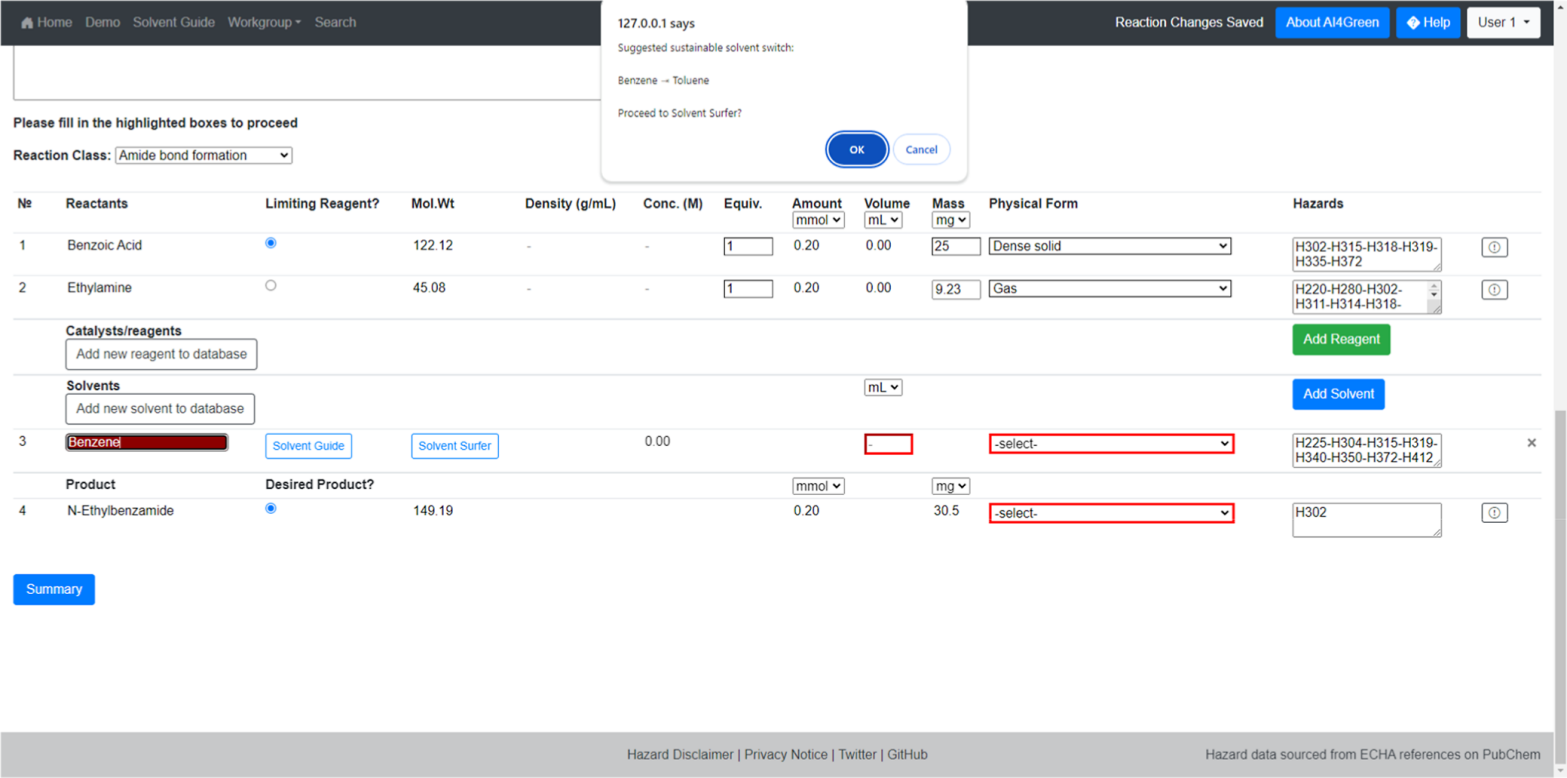
Integration of the Solvent Surfer into the AI4Green reaction constructor. The Surfer can be accessed by clicking on the “Solvent Surfer” button in the solvent entry. If there is a more sustainable solvent close by the chosen solvent, the switch will be suggested to the user.

The Solvent Surfer is accessible to AI4Green users via the homepage but can also be accessed easily during reaction design. A link to the Solvent Surfer is included as part of the AI4Green reaction constructor, which encourages users to consider any potential solvent substitutions when preparing to carry out a reaction. Furthermore, if a user selects a solvent that is close to a more sustainable option, they are alerted and prompted to check the Solvent Surfer to see if this is a viable switch (Figure 23). If a user has already selected a solvent and proceeds to the Solvent Surfer, the solvent and its suggested solvent table will be preloaded. This integration makes it easy to check for more sustainable solvents while minimizing the time needed. We anticipate that this design will have a positive impact upon sustainable chemistry during the reaction planning stage of chemical discovery.

## Conclusions and Future Work

This work describes the development and testing of interactive knowledge-based kernel PCA for the selection of solvent alternatives. An understanding of how the method could be used in this context has been developed through rigorous investigations into the robustness of the updated embeddings and the application of it to real world experimental data. This approach has been built-in to the latest sustainability plugin for AI4Green, the Solvent Surfer. This provides a simple intuitive interface that provides users with a quick and easy method to determine solvent alternatives and allows them to go beyond simple solvent selection by tuning solvent maps in response to real experimental data.

The Solvent Surfer has been proven to work in the context of experimental data taken from the literature, identifying potential alternatives for thioesterification and reductive amination reactions. In future, it would be of interest to use this tool alongside a solvent screen ab initio to provide a more realistic assessment of its utility. This would highlight any further benefits and limitations that are beyond the scope of this work and, more importantly, would provide a critical experimental validation of the alternatives identified by the Solvent Surfer. Additionally, other approaches, such as random forest or support vector machines could be investigated for clustering solvents, though this would require a considerably larger, labeled data set.

The current implementation of the Solvent Surfer contains only 57 solvents. Work to expand the default solvent list is ongoing, but this is challenging as the Kamlet–Abboud–Taft and Hansen parameters are not readily available for large numbers of solvents. An interface for users to add their own solvents could also be integrated into AI4Green, making the Solvent Surfer even more customizable. Additional functionality that allows users to track solvent usages and highlight any previously successful green substitutions could also be included.

## Data Availability

The data set and all associated code is hosted on the AI4Green GitHub at: https://github.com/AI4Green/AI4Green/tree/main/Webapp/sources/services/solvent_surfer and is publicly available. The data set and all references used for its construction is included as part of the Supporting Information.

## Supporting Information Available

Full solvent list, mathematical details and hyperparameters for interactive PCA, linear PCA of solvent data set, further details for cluster analysis (PDF)
Full Solvent data set with references (XLSX)
