# Supplementary material for: Interactive Knowledge-Based Kernel PCA for Solvent Selection
Source: ACS Sustain Chem Eng. 2025 Mar 14;13(11):4349–68. doi: 10.1021/acssuschemeng.4c07974 (PMC11938374; doi:10.1021/acssuschemeng.4c07974)
Supplement: Supplementary file 1 — sc4c07974_si_001.pdf [file sc4c07974_si_001.pdf]

Electronic Supporting Information

Interactive Knowledge-based Kernel PCA for Green Solvent Selection

Samuel Boobier[†], Joseph Heeley[†], Thomas Gärtner[‡], Jonathan D. Hirst[†*]

[†]School of Chemistry, University of Nottingham, University Park, Nottingham NG7 2RD, United Kingdom

[‡]Machine Learning Research Unit, TU Wien Informatics, Vienna 1040, Austria

* jonathan.hirst@nottingham.ac.uk

Number of pages: 13

Number of tables: 2

Number of figures: 6

Number of equations: 11

Full solvent dataset

The full dataset with references is provided alongside this document as a .csv file. The full data set as used in the deployed Solvent Surfer in AI4Green, is hosted on GitHub at: https://github.com/AI4Green/AI4Green/tree/main/Webapp/sources/services/solvent_surfer/data and is publicly available. The full list of solvents and their CHEM21 metrics are provided below.

The following solvents are missing Kamlet-Abboud-Taft parameters α, β and π* in the solvent dataset: Benzo trifluoride, 2-methoxy methanol, methyl cyclohexane, *tert*-butyl methyl ether, N,N-Dimethylethylene urea, N,N-Dimethylpropylene urea. To generate the PCA embeddings, these values were imputed as the mean value of the descriptor.

Table S1: List of solvents and their CHEM21 rankings.

Solvent	CHEM21		Solvent	CHEM21
1,2-Dichloroethane	Highly Hazardous		Hexafluoroisopropanol	No Ranking
1,2-Dimethoxyethane	Hazardous		Hexamethylphosphoramide	Highly Hazardous
2-Methoxyethanol	Hazardous		Hexane	Hazardous
Acetic acid	Problematic		i-Amyl alcohol	No Ranking
Acetic anhydride	Problematic		i-Butanol	No Ranking
Acetone	Recommended		Isopropyl alcohol	Recommended
Acetonitrile	Problematic		Isopropylacetate	Recommended
Anisole	Recommended		Methanol	Recommended
Benzene	Highly Hazardous		Methyl cyclohexane	Problematic
Benzotrifluoride	No Ranking		Methyl ethyl ketone	Recommended
Benzyl alcohol	Problematic		Methyl isobutyl ketone	Recommended
Carbon tetrachloride	Highly Hazardous		Methyl tert-butyl ether	Hazardous
Chloroform	Highly Hazardous		Methyl tetrahydrofuran	Problematic
Cyclohexane	Problematic		Dimethylethylene urea	No Ranking
Cyclohexanone	Problematic		Dimethylformamide	Hazardous
Cyclopentyl methyl ether	No Ranking		Dimethylpropylene urea	Problematic
Cyrene	No Ranking		Nitrobenzene	No Ranking
Dichloromethane	Hazardous		Nitromethane	Highly Hazardous
Diethyl ether	Highly Hazardous		N-methylpyrrolidinone	Hazardous
Dimethyl carbonate	No Ranking		Pentane	Hazardous
Dimethyl sulfoxide	Problematic		Propylene carbonate	No Ranking
1,4-Dioxane	Hazardous		p-Xylene	Problematic

Ethanol	Recommended		Pyridine	Hazardous
Ethyl acetate	Recommended		t-Butanol	Recommended
Ethyl lactate	No Ranking		Tetrahydrofuran	Problematic
Ethylene carbonate	No Ranking		Toluene	Problematic
Ethylene glycol	Recommended		Trifluoroethanol	No Ranking
Gamma-valerolactone	No Ranking		Water	Recommended
Heptane	Problematic			

Solving the optimisation problem

The combined optimisation problem incorporating control points as soft constraints[1] is given as

$$\alpha_s = \underset{\alpha \in \mathbb{R}^n}{\operatorname{argmax}}\ \alpha^T KWK\alpha + \rho(-\frac{1}{m}\left(\alpha^T K_{[:n,:m]}K_{[:m,:n]}\alpha - 2\boldsymbol{y}_s^T K_{[:m,:n]}\alpha\right)$$

$$\text{subject to} \qquad \alpha^T K\alpha = r^2$$

Combining and simplifying this expression gives the optimisation problem defined as a quadratic over an ellipsoid

$$\underset{\alpha \in \mathbb{R}^n}{\operatorname{argmax}}\ \alpha^T W'\alpha - 2\boldsymbol{b}^{\boldsymbol{T}}\alpha \tag{S1}$$

$$\text{subject to} \qquad \alpha^T K\alpha = r^2$$

where $W' = KWK - \frac{\rho}{m}K_{[:n,:m]}K_{[:m,:n]}$ and $\boldsymbol{b} = -\frac{\rho}{m}K_{[:n,:m]}\boldsymbol{y}_s$. This can be transformed to act over a hypersphere instead of a hyper ellipsoid by decomposing the positive-definite matrix K, introducing substitutions $\boldsymbol{v} = K^{\frac{1}{2}}\alpha$ and $\boldsymbol{d} = K^{-\frac{1}{2}}\boldsymbol{b}$ and replacing W' with the symmetric matrix $C = K^{-\frac{1}{2}}W'K^{-\frac{1}{2}}$

$$\underset{\boldsymbol{v} \in \mathbb{R}^n}{\operatorname{argmax}}\ \boldsymbol{v}^T C\boldsymbol{v} - 2\boldsymbol{d}^T\boldsymbol{v} \tag{S2}$$

$$\text{subject to} \qquad \boldsymbol{v}^T\boldsymbol{v} = r^2$$

This form of the optimisation problem can be solved using the corresponding Lagrange function (S3) and setting its derivatives equal to zero (S4)

$$\mathcal{L}(\boldsymbol{v}, \lambda) = \boldsymbol{v}^T C \boldsymbol{v} - 2\boldsymbol{d}^T \boldsymbol{v} - \lambda(\boldsymbol{v}^T \boldsymbol{v} - r^2) \qquad \text{(S3)}$$

$$C\boldsymbol{v} = \boldsymbol{d} + \lambda \boldsymbol{v}, \qquad \boldsymbol{v}^T \boldsymbol{v} = r^2 \qquad \text{(S4)}$$

Finding the solution to these two stationary constraints with the maximal value of λ gives the global optimum of equation (S2).[1–3] This can be done by decomposing the matrix $C = P\Delta P^T$ within the first constraint giving $P\Delta P^T \boldsymbol{v} = \boldsymbol{d} + \lambda \boldsymbol{v}$ which is then multiplied by the matrix P^T to give

$$\Delta \boldsymbol{t} = \widehat{\boldsymbol{d}} + \lambda \boldsymbol{t} \qquad \text{(S5)}$$

where $\boldsymbol{t} = P^T \boldsymbol{v}$ and $\widehat{\boldsymbol{d}} = P^T \boldsymbol{d}$. Rearranging this gives equation (S6), which can be substituted into the second stationary constraint from equation (S4) to give the secular equation (S7). The largest root of this equation provides the solution to equation (S2) and thus gives the optimal embedding.

$$t_i(\lambda) = \frac{\hat{\boldsymbol{d}}_i}{(\Delta_{ii} - \lambda)} \tag{S6}$$

$$g(\lambda) = \sum_i t_i^2(\lambda) - r^2 = 0 \tag{S7}$$

Efficient algorithm for updating embeddings

The roots of equation (S7) are computed with a complexity of $O(dn^3)$ for a d directional embedding with n samples and must be found for all directions at each user-interaction step. To provide a more efficient method to compute these embeddings, the quadratic term for a given direction can be calculated as a rank-one update of the same term for the previous direction. For the symmetric matrix C which defines the quadratic term for a direction s at interaction step k, the variance term $\bar{C}$ can be decomposed prior to any user interaction with a one-time complexity of $O(n^3)$. The first stationary constraint in equation (S4) for the direction $s + 1$ can be written as

$$C'\alpha = \lambda\alpha + \boldsymbol{a}_k \tag{S8}$$

where $\boldsymbol{a}_k$ represents the linear term for the control point constraints and C' is the rank-one update of the previous symmetric matrix C. Reusing the decomposition $C = P\Delta P^T$, which has already been computed, C' can be rewritten as

$$C' = C - \mu\boldsymbol{\alpha}_s\boldsymbol{\alpha}_s^T = P(\Delta - \mu zz^T)P^T \tag{S9}$$

where $\boldsymbol{\alpha}_s$ is the direction vector for direction s and $z = P^T \boldsymbol{\alpha}_s$. The selection or deselection of a control point also requires an update to the quadratic term of the optimisation, which can similarly be expressed as a rank-one update to matrix C.[1]

With the rank-one update to the diagonal matrix denoted as $\Theta = \Delta - \mu z z^T$, equation (S8) becomes

$$P\Theta P^T \alpha = \lambda\alpha + \boldsymbol{a}_k \Rightarrow \Theta\bar{\alpha} = \lambda\bar{\alpha} + \bar{\boldsymbol{a}} \qquad \text{(S10)}$$

where $\bar{\alpha} = P^T \alpha$ and $\bar{\boldsymbol{a}} = P^T \boldsymbol{a_k}$. The decomposition of this updated matrix $\Theta = \mathrm{V}\Delta'\mathrm{V}^T$ can be calculated with a complexity of $O(n^2)$, and can be used to express equation (S10) in the same form as equation (S5)

$$\Delta'\boldsymbol{t} = \lambda\boldsymbol{t} + \boldsymbol{f} \qquad \text{(S11)}$$

with $\boldsymbol{t} = V^T\bar{\alpha}$ and $\boldsymbol{f} = V^T\bar{\boldsymbol{a}}$. Combining this with the second stationary constraint in equation (S4) gives equation (S7), which can be used to compute the direction vector α_{s+1} as

$$\alpha_{s+1} = \bar{P}\left(\prod_{i=1}^{s} V_i\right) \cdot \boldsymbol{t}(\lambda_{max}), \quad \text{with} \quad \boldsymbol{t}_i(\lambda) = \frac{(\bar{P}\prod_{i=1}^{s} V_i)^T \boldsymbol{a}_k}{\delta_i^{s+1} - \lambda}$$

where $\bar{P}$ is the eigenvector matrix for the variance term $\bar{C}$ and δ_i^{s+1} is the eigenvalue of the quadratic term matrix for direction $s+1$. Solving this problem at interaction step k requires $O(d^2)$ matrix vector multiplications, each of which are of quadratic complexity $O(n^2)$. Therefore, the final complexity of this computation is $O(d^2 n^2)$.

Hyperparameters

Hyperparameters for the interactive kernel PCA can be viewed on GitHub in the cPCA class: https://github.com/AI4Green/AI4Green/blob/main/Webapp/sources/services/solvent_surfer/interactive/Embedder.py from line 115.

For the initial kernel PCA embedding, the hyperparameters are:

r = 3.0, slv_mode = "secular", sigma = None, epsilon = 0.5, degree = 1, const_nu = 1e-20, orth_nu = 5e3, precision = 2.56e-16, max_iters=800, parallelize = False

Linear PCA

A linear PCA of the dataset was conducted to provide a reference comparison for the kernel PCA. This was generated using the Scikit-Learn with default hyperparameters. A plot of the explained variance ratio for each principal component is shown in Figure S1, and the associated loadings for each descriptor are shown in Table S2. Plots showing the first three principal components for each of the case studies were also plotted to identify any potential trends in experimental data.

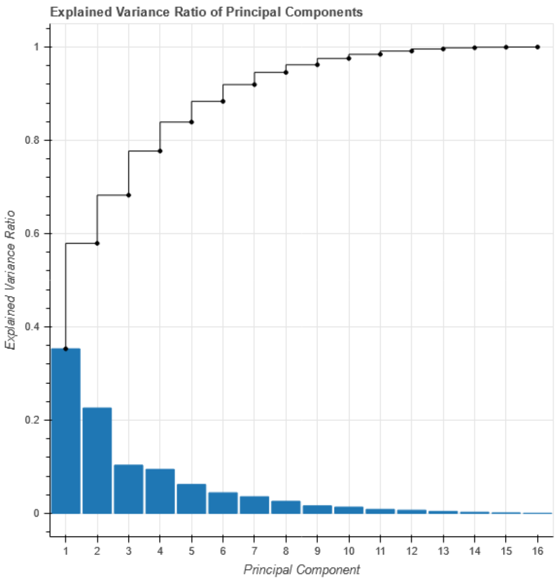

Figure S1: Explained variance ratio of principal components for linear PCA

Table S2: Principal component loadings of initial descriptors for linear PCA

Descriptor	PC1	PC2	PC3	PC4	PC5	PC6	PC7	PC8	PC9	PC10	PC11	PC12	PC13	PC14	PC15	PC16
Molecular Weight	-0.05	0.41	0.21	0.28	-0.40	0.08	-0.16	0.05	-0.05	-0.18	0.04	0.12	0.11	0.00	-0.26	0.62
Boiling Point	0.29	0.30	-0.17	0.10	-0.06	-0.11	0.29	0.12	0.12	-0.16	-0.24	-0.51	0.13	0.16	0.50	0.14
Density	0.17	0.18	0.61	0.10	-0.03	0.07	-0.19	-0.17	-0.28	-0.18	-0.37	0.10	0.05	-0.03	0.19	-0.43
Viscosity	0.19	0.07	-0.07	0.47	0.27	0.47	0.55	-0.23	-0.10	0.06	0.09	0.21	-0.07	-0.14	-0.05	0.02
Vapour Pressure	-0.22	-0.16	0.21	-0.24	-0.04	0.71	0.07	0.49	0.14	-0.02	-0.13	-0.11	0.14	0.05	0.06	0.03
Refractive Index	0.06	0.42	-0.16	-0.04	0.48	-0.04	-0.14	0.14	0.27	0.12	-0.39	0.08	0.28	0.01	-0.43	-0.10
LogP	-0.35	0.18	0.09	0.06	0.02	-0.24	0.22	0.27	0.00	0.39	-0.16	0.51	-0.13	0.15	0.41	0.10
Dipole Moment	0.34	0.10	-0.06	-0.16	-0.42	0.17	0.06	-0.10	0.13	0.35	-0.19	0.01	-0.45	0.42	-0.25	-0.12
Dielectric Constant	0.34	-0.06	0.04	-0.22	-0.11	-0.24	0.34	0.48	-0.18	-0.23	-0.14	0.16	-0.20	-0.45	-0.22	0.00
Alpha	0.09	-0.28	0.30	0.50	-0.08	-0.18	-0.02	0.25	0.10	0.52	-0.01	-0.34	0.13	-0.16	-0.15	-0.05
Beta	0.20	-0.07	-0.52	0.20	-0.08	0.22	-0.48	0.22	-0.43	0.15	-0.18	0.12	0.01	-0.12	0.21	0.01
Pi	0.37	0.08	0.12	-0.08	0.03	0.11	-0.28	-0.05	0.59	0.09	0.22	0.22	-0.12	-0.39	0.33	0.09
Dispersion	0.15	0.39	0.19	-0.12	0.33	0.03	-0.11	0.31	-0.32	0.15	0.57	-0.20	-0.20	0.18	0.00	-0.05
Polarity	0.36	-0.09	0.04	-0.27	-0.17	-0.04	0.16	-0.06	-0.13	0.21	0.22	0.27	0.71	0.18	0.03	0.01
H Bonding	0.23	-0.32	0.03	0.35	0.16	-0.09	-0.11	0.30	0.21	-0.42	0.08	0.27	-0.05	0.53	-0.02	-0.04
Molar Volume	-0.21	0.32	-0.23	0.21	-0.42	0.02	0.07	0.19	0.18	-0.13	0.30	0.06	0.14	-0.11	-0.03	-0.60

Thioesterification

The linear PCA shows good correlation to the experimental thioesterification data for low yielding solvents but fails to accurately cluster the high yielding solvents. This is similar to the initial kernel PCA embedding, which can be improved using the interactive approach.

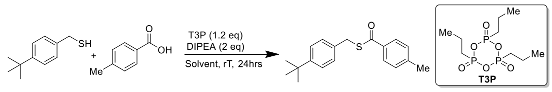

Solvent	CHEM21 Ranking	% Conversion
EtOAc	Recommended	40
2-MeTHF	Problematic	42
CPME	No Ranking	46
DMC	No Ranking	55
DCM	Hazardous	77
MeCN	Problematic	77
DMF	Hazardous	79

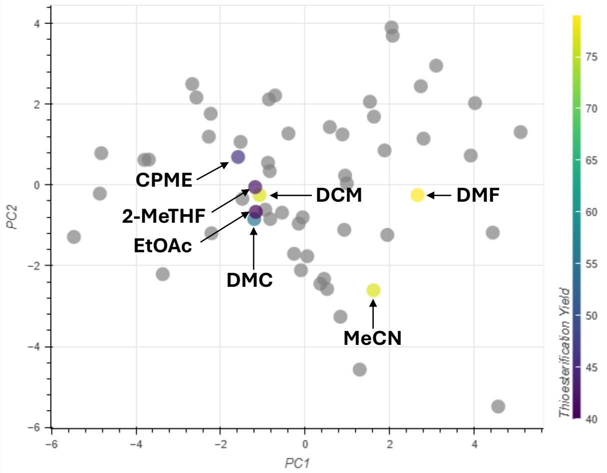

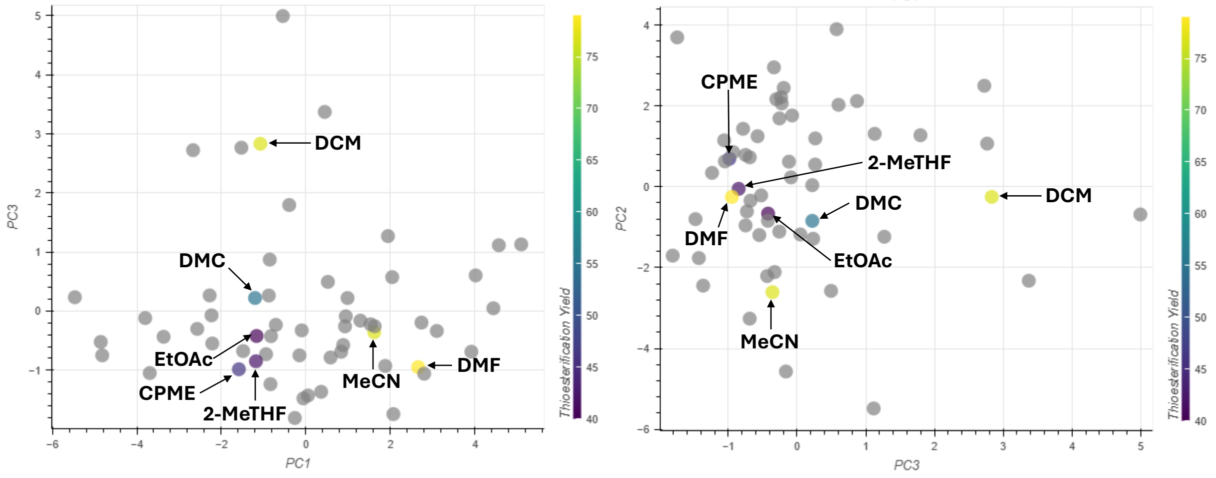

Figure S2: Linear PCA of the solvent dataset, coloured by yield according to the thioesterification data of case study 1.

Reductive Amination

The linear PCA shows good correlation to the experimental reductive amination data for mid yielding solvents, though these are more spread out than in the initial kernel PCA projection. The Both the high and low yielding solvents are not clustered according to the experimental data. This too is true in the initial kernel PCA, but can be addressed through the interactive approach.

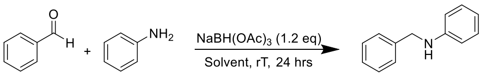

Solvent	CHEM21 Ranking	% Conversion
DCM	Hazardous	100
DCE	Highly Hazardous	100
IPA	Recommended	100
CPME	No Ranking	86
DMC	No Ranking	85
TBME	Hazardous	85
EtOAc	Recommended	80
2-MeTHF	Problematic	80
DMF	Hazardous	39
THF	Problematic	29

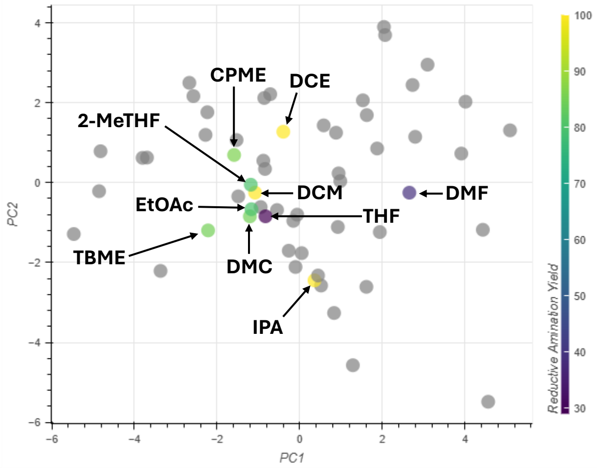

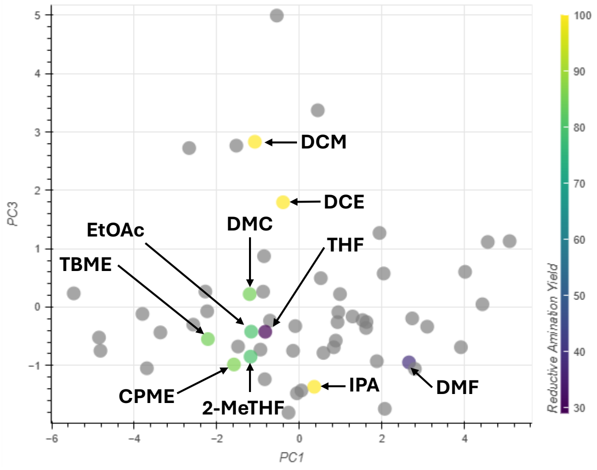

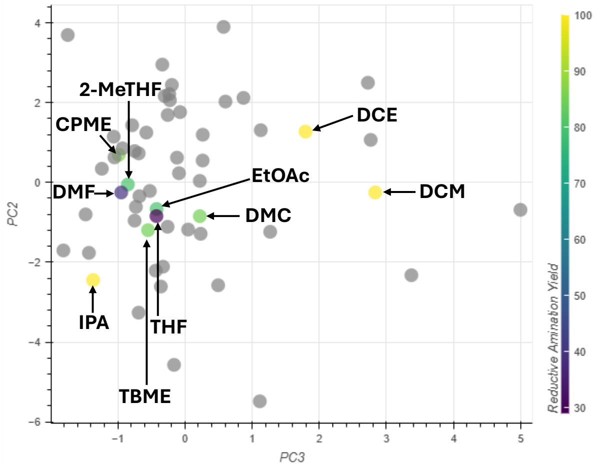

Figure S3: Linear PCA of the solvent dataset, coloured by yield according to the reductive amination data of case study 2.

Solubility of Paracetamol

The linear PCA gives reasonably good clustering of high and mid yielding solvents, thought there are a few outliers. The initial kernel PCA projection gives similar performance. The use of interactive PCA in this scenario can help to separate the high and mid yielding domains.

Solvent	CHEM21 Ranking	Solubility (g 100g⁻¹)
Hexane	Hazardous	0.0007
Cyclohexane	Problematic	0.005
Benzene	Highly Hazardous	0.02
THF	Problematic	0.03
Toluene	Problematic	0.03
$CHCl_3$	Highly Hazardous	0.04
Anisole	Recommended	0.05
^{i}PrOAc	Recommended	0.75
EtOAc	Recommended	0.94
H_2O	Recommended	1.49
MIBK	Recommended	1.66
MeCN	Problematic	2.75
1,4-dioxane	Hazardous	5.56
Acetone	Recommended	9.94
IPA	Recommended	12.11
EtOH	Recommended	20.99
MeOH	Recommended	33.21

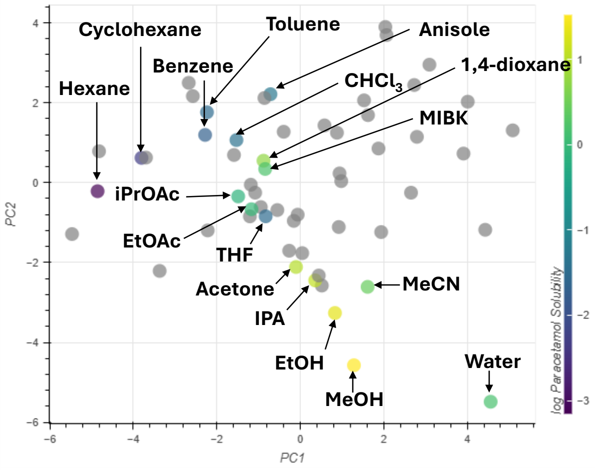

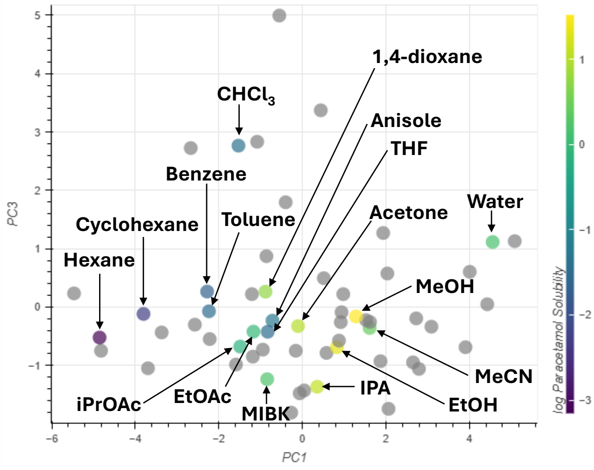

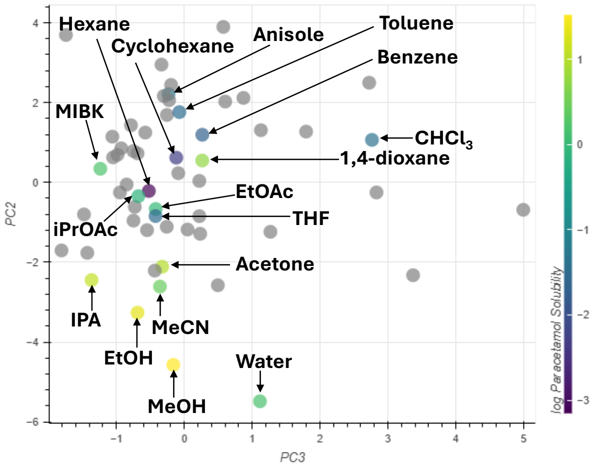

Figure S4: Linear PCA of the solvent dataset, coloured by solubility according to the paracetamol solubility data of case study 3.

Clustering

To find the optimal value of k, KMeans clustering was carried using the implementation in Scikit-Learn using random_state=42 and was applied only to the kernel PCA. The within-cluster sum of squares (WCSS) score was calculated for multiple values of k between 1 – 10. The subsequent elbow plot (Figure S5a) shows the optimal value as k=5. Figure S5b shows the clusters of the kernel PCA where k=5.

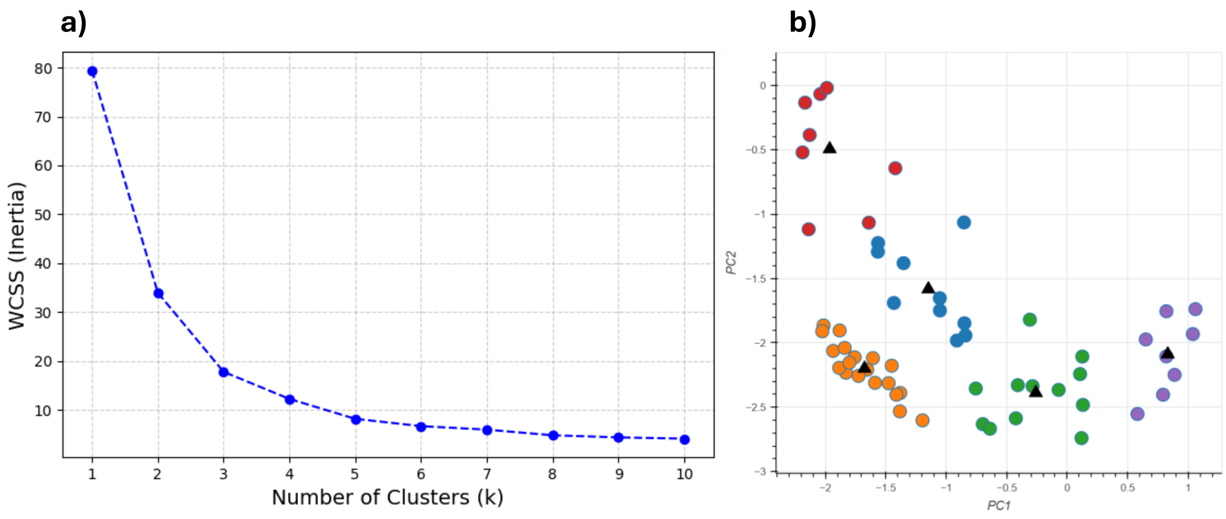

Figure S5: **a)** within-cluster sum of squares vs number of KMeans centroids, **b)** clusters plotted in kernel PCA for k=5. Points are colour-coded according to clusters and centroids are denoted by triangles.

Cluster Stability

To assess cluster stability, the kernel PCA embedding was subject to 10 runs of KMeans clustering varying the random_state parameter between 0 – 9. The Adjusted Rand Index (ARI) between each run was calculated and the mean and standard deviation was taken as the final stability score. The ARI was calculated using the implementation in Scikit-Learn using default parameters. For the initial embedding, this gave a mean of 0.74 and a standard deviation of 0.13. This procedure was carried out for each embedding after movement of a control point.

Paracetamol solubility case study

The movements of 1,4-dioxane and THF from embedding 4 of paracetamol solubility case study are shown in Figures S6a and S6b respectively. Moving 1,4-dioxane to the mid solubility domain has little effect on the positions of other solvents, while movement of THF into the low solubility domain removes the boundary between low and mid solubility domains.

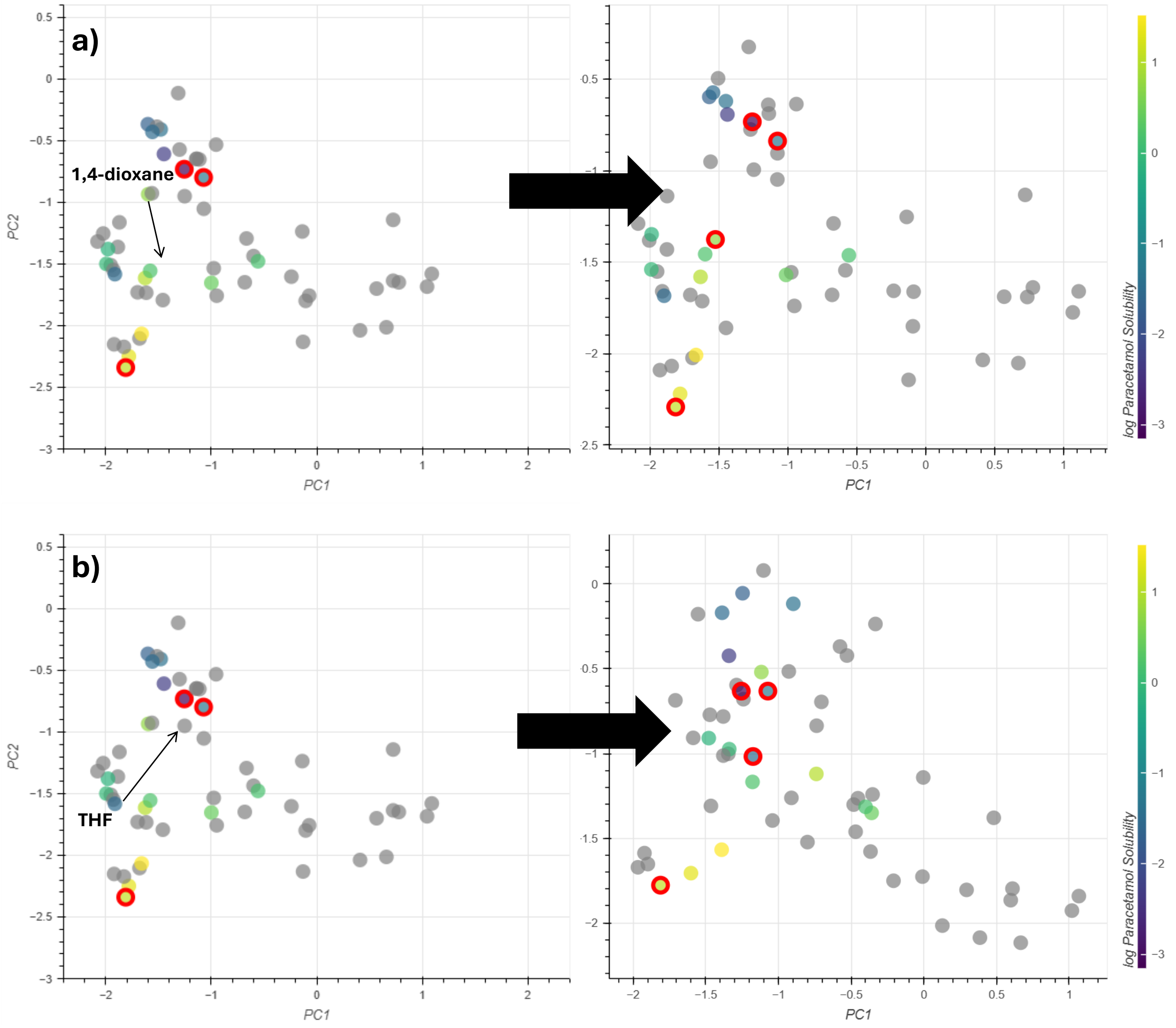

Figure S6: a) Movement of 1,4-dioxane to mid solubility domain, **b)** movement of THF to the low solubility domain.

References

(1) Oglic, D.; Paurat, D.; Gärtner, T. Interactive Knowledge-Based Kernel PCA. In *Machine Learning and Knowledge Discovery in Databases*; Calders, T., Esposito, F., Hüllermeier, E., Meo, R., Eds.; Springer Berlin Heidelberg: Berlin, Heidelberg, 2014; pp 501–516.

(2) Forsythe, G. E.; Golub, G. H. On the Stationary Values of a Second-Degree Polynomial on the Unit Sphere. *Journal of the Society for Industrial and Applied Mathematics* **1965**, *13* (4), 1050–1068.

(3) Gander, W. Least Squares with a Quadratic Constraint. *Numer. Math.* **1980**, *36* (3), 291–307.
